# Comprehensive Characterization of Mesenchymal Stem Cells from Human Placenta and Fetal Membrane and Their Response to Osteoactivin Stimulation

**DOI:** 10.1155/2012/658356

**Published:** 2012-06-04

**Authors:** C. M. Raynaud, M. Maleki, R. Lis, B. Ahmed, I. Al-Azwani, J. Malek, F. F. Safadi, A. Rafii

**Affiliations:** ^1^Stem Cell and Microenvironment Laboratory, Weill Cornell Medical College in Qatar, Qatar Foundation, Doha 24144, Qatar; ^2^Departments of Genetic Medicine and Obstetrics and Gynecology, Weill Cornell Medical College, New York, NY 10065, USA; ^3^Maternal and Fetal Unit, Women Hospital, Hamad Medical Corporation, Doha 3050, Qatar; ^4^Genomic Core, Weill Cornell Medical College in Qatar, Qatar Foundation, Doha 24144, Qatar; ^5^Departments of Anatomy and Cell Biology, Orthopaedic Surgery and Sport Medicine, Temple University School of Medicine, Philadelphia, PA 19140, USA; ^6^Department of Cell and Developmental Biology, Weill Cornell Medical College, Doha 24144, Qatar

## Abstract

Mesenchymal stem cells (MSCs) are the most promising seed cells for cell therapy and can be isolated from various sources of human adult tissues such as bone marrow (BM-MSC) and adipose tissue. However, cells from these tissues must be obtained through invasive procedures. We, therefore, characterized MSCs isolated from fresh placenta (Pl-MSC) and fetal membrane (Mb-MSC) through morphological and fluorescent-activated cell sorting (FACS). MSC frequency is higher in membrane than placenta (2.14%  ± 0.65 versus 15.67%  ± 0.29%). Pl/Mb-MSCs *in vitro* expansion potential was significantly higher than BM-MSCs. We demonstrated that one of the MSC-specific marker is sufficient for MSC isolation and that culture in specific media is the optimal way for selecting very homogenous MSC population. These MSCs could be differentiated into mesodermal cells expressing cell markers and cytologic staining consistent with mature osteoblasts and adipocytes. Transcriptomic analysis and cytokine arrays demonstrated broad similarity between placenta- and membrane-derived MSCs and only discrete differences with BM-MSCs with enrichment of networks involved in bone differentiation. Pl/Mb-MSCs displayed higher osteogenic differentiation potential than BM-MSC when their response to osteoactivin was evaluated. Fetal-tissue-derived mesenchymal cells may, therefore, be considered as a major source of MSCs to reach clinical scale banking in particular for bone regeneration.

## 1. Introduction

Multipotent mesenchymal stem cells (MSCs) are able to self-renewed and differentiate into mesodermal lineages such as adipogenic, chondrogenic, osteogenic, myogenic, and angiogenic cells [1]. MSCs were initially isolated from bone marrow by Haynesworth et al. [2]. In the bone marrow, they provide support for hematopoiesis [3]. They also secrete several growth factors important in angiogenesis including vascular endothelial growth factors [4]. Therefore, they represent one of the most promising cell types for cell therapies and tissue engineering or trauma repair. Indeed, different preclinical experiments using MSCs have been performed demonstrating their ability to improve myocardial or cerebral function after ischemic stress, or liver and joint damage after traumatic or surgical injuries [5–8]. They might also be optimal for cellular therapy by inducing immune tolerance. Indeed, they can generally be transplanted even in large outbreed animals across major histocompatibility complex (MHC) barriers without need for immune suppression [9]. 

The bone marrow is the traditional source of human MSCs, but they have been isolated from a wide variety of human adult tissues such as adipose tissue [10], lung [11], and liver [12]. However, cells from most of these tissues must be obtained through invasive procedures, and the interindividual variability is hard to control. Several studies describe the isolations of MSCs from fetal tissues such as umbilical cord blood [13], placenta [14–16], amniotic membrane [17, 18], and amniotic fluid [19], and they have described their MSCs characteristics.

Osteoactivin (OA) has the ability to regulate cell proliferation, adhesion, differentiation, and synthesis of extracellular matrix proteins in various cell types [20–30]. OA messenger ribonucleic acid (mRNA) and protein are expressed by human and rodent osteoblasts [29, 30]. OA down-regulation decreases osteoblast differentiation and function [31]. Osteoblast cells express increasing levels of OA protein during their differentiation. OA has been demonstrated as essential for the differentiation and functioning of osteoblast cells [32]. We previously demonstrated that OA induces similar osteoblastic differentiation than BMP2 in mice MSC suggesting that OA may be a novel osteoinductive agent [29, 31, 32].

In this study, we optimized the isolation of placental and amniotic membrane MSC and compared their proliferative and differentiation potential to BM-MSCs. We isolated through different methods MSCs from placenta and fetal membranes, and we qualified them according to the standardize protocols from the international society for cellular therapy (ISCT) [33]. We further investigated and demonstrated that OA triggers osteoblastic differentiation in human MSCs and that the differentiation was even more important in fetal MSCs as compared to BM-MSCs. We illustrate that fetal tissues derived MSCs are more prone than BM-MSCs to differentiate into osteoblasts.

## 2. Materials and Methods

### 2.1. Placenta and Fetal Membranes Collection

Following approval from the Internal Review Board (HMC-IRB Protocol 9109/09, Weill Cornell Medical College in Qatar), placentas and fetal membranes were collected from donors at Woman's Hospital at Hamad Medical Corporation immediately after elective caesarean section at term in the absence of labor, preterm rupture of membrane, chorioamnionitis, preeclampsia, intrauterine growth retardation, or chromosomal abnormalities. The specimen were completely deidentified and considered as biological waste. Therefore, no consent form was taken from the patients.

### 2.2. Mesenchymal Stem Cell Isolation

Supplementary Figure 1 (available at doi:10.1155/2012/658356) depicts the isolation procedures used in this study. For placenta, the decidua basalis was removed prior to harvesting the placental tissues. The placenta parts were free of any fetal membrane. For fetal membrane, we decided not to separate the amnion and chorion parts to illustrate the most direct workflow for MSCs isolation. The harvested pieces of tissues were washed in phosphate buffer saline (PBS, PH 7.4), mechanically minced into pieces of approximately 1 mm^2^, and subsequently digested with dispase (1 mg/mL, Hyclone), collagenase (300 U/mL) (Hyclone), hyalluronidase (100 U/mL, Hyclone), and DNAse I (80 U/mL, Roche) for 1 h at 37°C under agitation (150 rpm). The homogenate was subsequently washed in PBS. Cells were then filtered on a 100 *μ*m cells strainer. Red blood cells and aggregates were eliminated on a Ficoll gradient. The mononucleated fraction was collected for further analysis.

Two million viable cells were then either directly plated in MSCs culture media (DMEM low glucose with 20% FBS, 2 mM L-Glutamin, and 1% penicillin/streptomycin [16]) or sorted through SORP FACS Aria II (BD Biosciences) and then plated in the MSC culture media in 24 well plates. Cultures were incubated in humidified 5% CO_2_ incubators and the media was replaced every 3 days.

Several bone marrows MSCs from different donors (BM-MSCs) were purchased from Stem Cell Inc. (number MSC-001F, Stem Cell Inc.) and PromoCell (number 12974, PromoCell) and maintained in the same culture conditions as placenta-/membrane-derived MSCs (Pl/Mb-MSCs). We performed all analysis at the 4th passages in order to obtain a homogenous cell population and sufficient number of cells to perform all analysis in parallel.

### 2.3. Immunostaining and Fluorescence-Activated Cell Sorting (FACS) Analysis

For flow analysis of cell surface antigens and cell sorting, MSCs were stained for the expression of CD45, CD34, CD73, CD105, CD90, and CD29 using Mouse anti-human CD45 antibody (BD Biosciences, number 339192, clone 2D1) coupled with Amcyan, Mouse anti-human CD34 (BD Biosciences, number 555821, clone 581) coupled with FITC, Mouse anti-human CD105 (biolegend, number 323212, clone 43A3) coupled with AF647, Mouse anti-human CD73 (BD Biosciences, number 550257, clone AD2) coupled with PE, Mouse anti-human CD29 (biolegend, number 323212, clone TS2/16) coupled with APC-Cy7, and Mouse anti-human CD90 (BD Biosciences, number 550402, clone 5E10) coupled with AF700.

 Briefly, 1.10^6^ cells were harvested and nonspecific sites were blocked in PBS-5%FBS-1%BSA-10%FcR Blocking Reagent (Myltenyi Biotec) for 30 minutes on ice. Cell suspension was incubated with specific antibodies for 45 minutes on ice. After washes in PBS and filtration on 45 *μ*m strainer, cells were analyzed by fluorescence activated-cell sorting (FACS) on a SORP FACSAriaII (BD Biosciences) as described later. Data were processed with FACSDiva 6.3 software (BD Biosciences). Doublets were excluded by FSC-W × FSC-H and SSC-W × SSC-H analyses, single-stained channels were used for compensation, and fluorophore minus one (FMO) controls were used for gating, 500 000 events were acquired per sample [34].

### 2.4. Immunocytochemistry

Cells in culture were grown on 8 chamber slides (BD falcon, number 354102) and stained as follows.

The antibodies used were Mouse anti-human CD29-FITC (biolegend, number 303016, clone TS2/16), CD73-PE (BD Biosciences, number 550257, clone AD2), CD90-AF568 (BD Biosciences, number 550402, clone 5E10), CD34-PE (BD Biosciences, number 555822, clone 581), CD45-Amcyan (BD Biosciences, number 339192, clone 2D1), unconjugated, and CD105 (BD Biosciences, number 555690, clone 266) revealed by a secondary goat anti mouse IgG1 antibody (invitrogen, number A-21121).

Briefly, nonspecific sites and Fc receptors were blocked with PBS/0.3% bovine serum albumin/0.5% HS for 30 minutes and FcR blocking reagent (Miltenyi, number 120-000-442). Sections were incubated with primary antibodies (1 hour 30 minutes), washed twice in PBS/0.5% Tween 20 (Sigma-Aldrich), and if necessary incubated with secondary antibodies (1 hour, AF488 goat anti-mouse IgG1 at 0.5 *μ*g/mL). Nuclei were counterstained with 4-,6-diamidino-2-phenylindole (Invitrogen). Slides were mounted with the Fluoromount Kit (Invitrogen). Sections were analyzed with a Zeiss confocal microscope Laser Scanning Microscope 710 (Carl Zeiss). Pictures were analyzed with Zen 2008 V5,0,0228 software (Carl Zeiss).

### 2.5. Mesodermal Lineage Differentiation

#### 2.5.1. Adipogenic Lineage

Adipogenic differentiation was induced by culturing 80% confluent MSC for 3 weeks in DMEM-HG, 1 *μ*M dexamethasone, 5 *μ*g/mL insulin (Sigma), 60 *μ*M indomethacin (Sigma; catalogue number: 17378-5G), and 0.5 mM 3-Isobutyl-1-methylxanthine (IBMX; Sigma; catalogue number: I5879) [35]. Adipogenic differentiation was assessed by staining cells in wells with Oil Red O.

#### 2.5.2. Osteogenic Lineage

Osteogenic differentiation was induced by culturing 90% confluent MSC for 3 weeks in DMEM-LG, 10% FCS, 0.1 *μ*M dexamethasone (Sigma; Australia Register Number: 16375; Melbourne, Victoria, Australia), 50 *μ*M L-ascorbic acid-2-phosphate (Sigma; catalogue number: A8960-5G; Castle Hill, New South Wales, Australia), 10 mM *β*-glycerol phosphate disodium salt pentahydrate (Sigma; catalogue number: 50020), and 0.3 mM inorganic (sodium) phosphate (Sigma) [35, 36]. Osteogenic differentiation was assessed by staining with Alizarin Red S.

#### 2.5.3. Osteoactivin Stimulation

Three days after MSCs plating (50% confluence), recombinant human osteoactivin was delivered in a single dose of 100 ng/mL in differentiation media DMEM-LG, 10% FCS, 0.1 *μ*M dexamethasone, 50 *μ*M L-ascorbic acid-2-phosphate, 10 mM *β*-glycerol phosphate disodium salt pentahydrate, and 0.3 mM inorganic (sodium) phosphate. Controls were carried out in regular cell culture media described above. The osteogenic differentiation was then assessed at day 7, 14, and 21 after OA treatment by staining with Alizarin Red S. Following treatment, color phase contrast microscopy pictures were acquired at different time points. Analysis of the red channel was performed using image J (NIH). Normalization for cell number was done using the blue channel.

### 2.6. Transcriptomic Analysis

RNA was isolated using Trizol reagent followed by additional purification using RNAeasy extraction kit from Qiagen (QIAGEN, number 74106) with RNA yields that produces satisfactory microarray data. Two quality control measures were carried out: (1) a spectrophotometric analysis and (2) a size fractionation procedure using a microfluidics instrument (Agilent Technologies). 200 ng of total RNA were analyzed on Affymetrix GeneChip Human Genome U133 Plus 2.0 Array. Data were analyzed using Parteck Software (V6.09.1110-6; Affimetrix). Class comparison between BM-MSCs and Pl/Mb-MSCs (three biological replicates of each) was performed to identify gene expression changes with a significant expression differences (*P* < 0.05) and 2-fold increase or decrease expression. Parteck Software gene ontology tools were used to determine gene enrichment [37].

We used Ingenuity Pathway Analysis software (Ingenuity Systems, Redwood City, CA) to identify and analyze relevant pathways from the gene lists obtained after comparison of BM-MSC and Mb/Pl-MSC. Networks were constructed by overlaying the genes in the gene list onto a global molecular network developed from information contained in the Ingenuity Pathways Knowledge database using keywords such as organ formation and osteoblast differentiation. Networks of the genes up- or downregulated Pl/Mb-MSCs as compared to BM-MSCs were then algorithmically generated based on their connectivity. A network is a graphical representation of the molecular relationships between genes. Genes are represented as nodes, and the biological relationship between two nodes is represented as a line. All edges are supported by at least one reference from the literature, from a textbook, or from canonical information stored in the Ingenuity Pathways knowledge database. *P* values for the enrichment of canonical pathways were then generated based on the hypergeometric distribution and calculated with the right-tailed Fisher's exact *t*-test for 2 × 2 contingency tables.

### 2.7. Phospho-Kinase Array

MSCs were cultivated in differentiation or control media in presence or absence of OA for 4 h. Cells were harvested and proteins extracted as recommended and quantified based on sample absorbance at 280 nm using nanodrop device (Thermo-Scientific). 200 *μ*g of protein was loaded on R&D system Human Phospho-kinase Antibody Array (R&D system, number ARY003) according to manufacturer's instructions.

Arrays were revealed using HorseRadish Peroxidase (HRP) and SuperSignal West Pico Chemiluminescent Substrate (Thermo Scientific). Data were collected using Geliance CCD camera (Perkin Elmer) and extracted using Image J software (NIH). Briefly, arrays' pictures were inverted and background subtracted. We defined a 120-micron diameter area for signal capture. Median pixel density was used to evaluate the signal. For comparison, independent array values were normalized on their positive control intensity values.

### 2.8. Cytokine Array

MSCs were cultivated in serum free media for 72 hours as previously published [38]. Supernatant was collected and proteins quantified based on sample absorbance at 280 nm using nanodrop device (Thermo-Scientific). 200 *μ*g of protein was loaded on R&D system Human Cytokine Antibody Array panel A (R&D system, number ARY005) according to manufacturer's instructions. Arrays were analyzed as described above.

### 2.9. Statistical Analysis

Student-*t*, Fisher exact, or chi-square tests were performed as appropriate. All *P*-values are two-sided with statistical significance evaluated at the 0.05 alpha level. All statistical analysis were done using the data analysis plug-in shipped into the Excel 2008 for Mac (Microsoft). We first calculated the variance of two paired. Mean ± SEM are shown on the graphs. All results are representative of the indicated number of independent experiments.

## 3. Results

### 3.1. MSCs Isolation Methods

Supplementary Figure 1 depicts the workflow chart of the enzyme-mediated cell isolation of human term placenta/membrane, by direct culture or cell sorting, for derivation of fibroblast-like cells, that we characterized as multipotent mesenchymal stem cells (MSCs). Two methods were used to isolate MSCs. (i) selection in specific MSC media after direct culture of cell suspension obtained following tissue digestion, (ii) fluorescent-activated cell sorting according to expression of specific MSCs markers as defined by the international society for cellular therapy (ISCT, positive for CD105, CD73, CD90, CD29, and negative for CD45 and CD34) [39]. 15 different amniotic membranes and placentas were used in this study. We were able to differentiate MSCs from all of these specimens, all analyses are representative of 3 different samples.

### 3.2. MSCs Are More Abundant in Membrane Than Placenta

**Figure 1:**
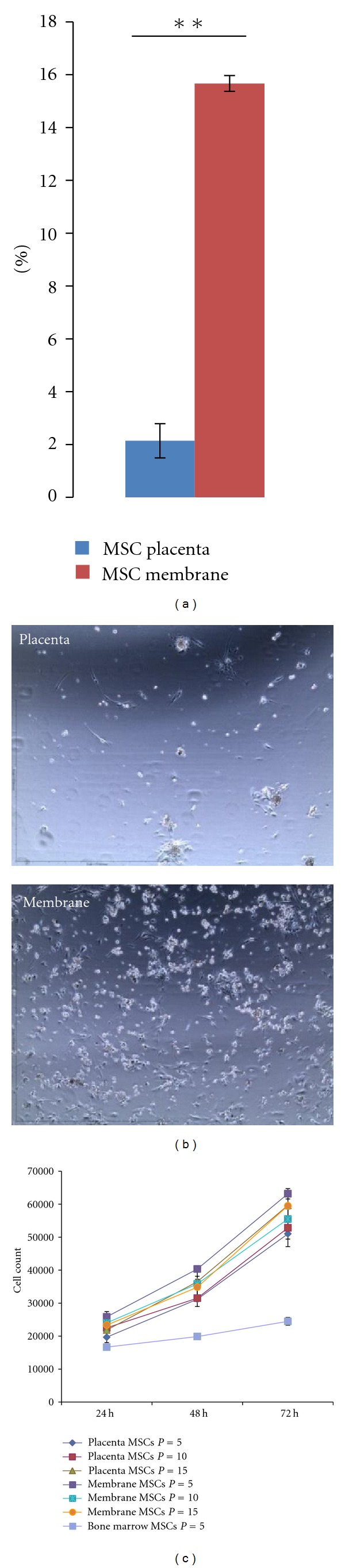
Higher proportion of MSCs can be isolated from the membrane compare to the placenta. Fetal tissue derived MSCs display greater proliferation kinetic than bone marrow derived MSCs. (a) MSCs were defined as CD45^−^, CD34^−^, CD29^+^, CD90^+^, CD73^+^, and CD105^+^. Their proportion was then calculated in freshly digested placenta specimens and fetal membranes form 3 different donors. MSCs represented 15.67% (±0.29%) and 2.14% (±0.65%) of cells isolated from the membrane and placenta, respectively (**P = 9.25 · 10^−4^). (b) Day 1 phase contrast microscopy of adherent cells from placenta and membrane directly plated after tissue digestion in MSCs media. We can see significantly more adherent cells from digested membrane compared to placenta. (c) MSCs derived from placenta and membranes were expanded up to passage 15 without changes of their morphology or proliferation rate. Proliferation rate was assessed by cell counting at different passages. It was similar in placenta- and membrane-derived MSCs with no differences between early and late passages. However, proliferation rate was significantly higher than proliferation rate of bone-marrow-derived MSCs.

We quantified the number of MSCs in fetal membrane and placenta using polyvariate flow cytometry. CD45^−^, CD34^−^ cells were selected and analyzed for the expression of CD105, CD73, CD90, and CD29. MSCs as defined by these 4 markers were significantly more abundant in the membrane, Mb-MSC 15.67% (±0.29%) than the placenta, Pl-MSC 2.14% (±0.65%, Figure 1(a)).

The same results were found when 2 million cells were directly plated after tissue digestion. The number of adherent cells was significantly higher at day 1 in the membrane compare to the placenta (Figure 1(b)).

### 3.3. Placenta or Membrane Isolated MSCs Have Greater Proliferation Ability Than Bone Marrow MSCs

Growth kinetic of Mb-MSCs and Pl-MSCs were compared with BM-MSCs at the same passage. The proliferation rate of Pl/Mb MSCs was significantly higher than BM-MSCs (Figure 1(c)). Moreover, Mb-MSCs and Pl-MSCs were expandable up to passage 15 without modification of their morphology or proliferation rate as described in other studies however BM-MSC stopped proliferating after passages 7 to 8 [14].

### 3.4. Analysis of Subpopulations Based on CD90 and CD29 Expression

**Figure 2:**
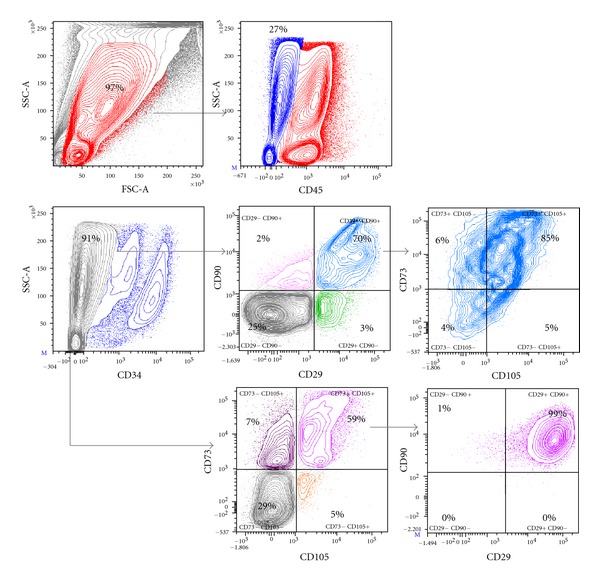
The Cell analysis strategy allows the isolation of 4 different cell populations. Cells were stained with mouse anti human CD45-Amcyan, CD34-FITC, CD29-APC-CY7, CD90-AF700, CD73-PE, CD105-AF647. After FSC-A/SSC-A selection, only CD45^−^ and CD34^−^ cells were considered. CD73, CD105, CD90, and CD29 profiles were then analyzed. CD73^+^ CD105^+^ cells were more than 85% to 98% positive for CD90 and CD29 (lower panel). However, CD90^+^ CD29^+^ represented a more heterogenous population when looking at CD73, CD105 stainings. We defined different cell populations based on CD90 and CD29 subpopulation: CD90^+^ CD29^+^; CD90^−^ CD29^+^; CD90^+^ CD29^−^; CD90^−^ CD29^−^ (middle panel).

We first defined our cell population as being negative for CD45 and CD34. In all our independent experiences, the vast majority if not all (85% to 99%) of CD73^+^, CD105^+^ cells was also expressing CD90 and CD29 reaching the canonical definition of MSCs [33, 39]. However, the number of CD90^+^, CD29^+^ cells positive for CD73 and CD105 was lower ranging from 65% To 85% (Figure 2). We, therefore, decided to further analyze the populations characterized by CD90/CD29 expression. We wondered our ability to derive MSCs from these different cell populations. We sorted 4 subpopulations based on the expression of those markers: CD90^+^ CD29^+^; CD90^−^ CD29^+^; CD90^+^ CD29^−^; CD90^−^ CD29^−^ in placentas and fetal membranes (Figure 2 and Supplementary Figure 2). We performed all experiences on 3 independent donors. The purity of the sort was assured by applying the purity mask and controlling for the purity of the different cell populations sorted.

None of the CD90^−^ CD29^−^ cells were able to grow in MSCs media. The CD29^−^ CD90^−^ is actually a very homogenous population containing mostly CD73^−^ CD105^−^ cells (Supplementary Figure 2). In contrast, we were able to derive mesenchymal like cells from the 3 other sorted subpopulations. This indicates that expression of at least one of these 2 markers is indispensable for MSCs isolation and qualification.

After 4 passages, the large majority of the cells sorted expressed CD90 and CD29. (Table 1 and Supplementary Figure 3). At this stage, the large majority displayed a CD73^+^ CD105^+^ profile. We confirmed the expression of all markers by immunofluorescence staining (Supplementary Figure 4).

### 3.5. Differentiation Assay of Placenta and Membrane MSCs

**Figure 3:**
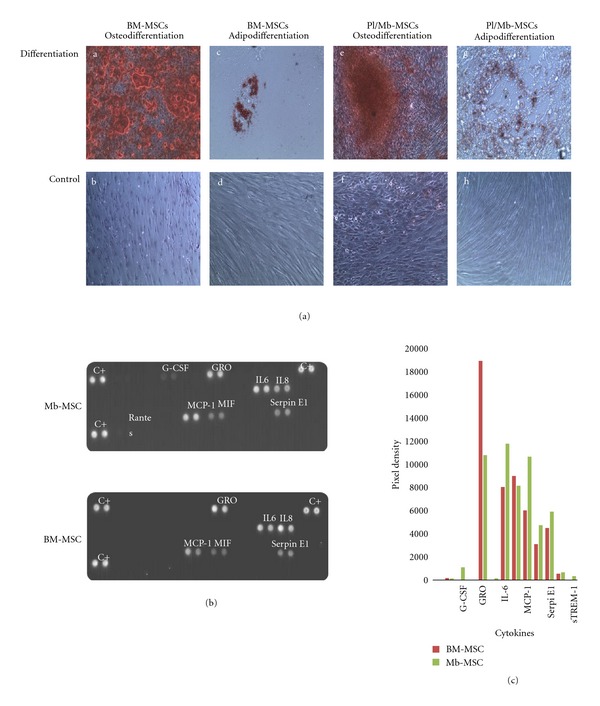
Differentiation assay of Pl/Mb-MSCs in comparison to BM-MSCs and cytokines expression. (a) Representative differentiation of Pl/Mb-MSCs passage 4 is shown. Cells were kept in induction medium (differentiation) or control standard medium (control). (a–d) Osteogenic and adipocyte differentiation and control for BM-MSCs. (f–h) Osteogenic and adipocyte differentiation and control for Mb-MSCs. (b) Cytokine expressions of Mb-MSCs and BM-MSCs using the proteome profiler. (c) Quantification of cytokine optical density. Measurements were obtained with image J software (NIH).

Specific induction of adipogenic and osteogenic differentiation was performed on Mb/Pl MSCs sorted based on CD29 and CD90 expression or directly plated after isolation and compared to BM-MSCs differentiation (Figure 3(a)).

All different cell populations from placenta or membrane regardless of the isolation protocol were identically able to differentiate into adipocytes and osteoblasts confirming their phenotypic and functional similarity (data not shown).

### 3.6. Cytokines Secretion of MSCs

 Cytokine secretion profile was highly similar between fetal and BM-MSCs with strong secretion of GRO*α* (CXCL1), IL-6, IL-8 (CXCL8), MCP-1 (CCL2), MIF (GIF, DER6), and serpin E1 (PAI-1), see Figures 3(b) and 3(c). Only discrete differences were noted, GRO*α* (CXCL1) secretion by the BM-MSCs was higher than its expression in the fetal MSCs, whereas the expression of IL-6 and MCP-1 in the BM-MSCs was comparatively lower than in fetal MSCs.

### 3.7. Transcriptomic Comparison of Fetal and Bone Marrow MSCs

**Figure 4:**
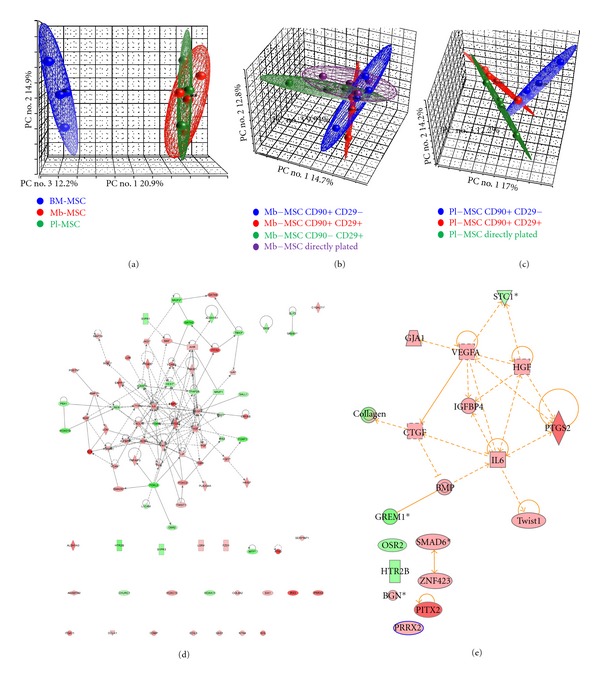
PCA representation of transcriptomic comparison of Pl-MSCs, Mb-MSCs and BM-MSCs and ingenuity pathways analysis. (a) Mb-MSCs (red) and Pl-MSCs (green) are overlapping clearly indicating the impossibility to differentiate these MSCs at a transcriptional level. They can clearly be differentiated from bone marrow MSCs (blue). (b) The PCA overlapping between every cell sorted sub-population from membrane (CD29^+^ CD90^+^ red; CD29^−^ CD90^+^ blue; CD90^−^ CD29^+^ green) or nonsorted cells (Mb-MSCs purple) indicate the impossibility to discriminate the different MSCs subpopulation at a transcriptional level. (c) PCA of different MSC subpopulation from placenta (CD29^+^ CD90^+^ red; CD29^−^ CD90^+^ blue) or directly isolated cells (Pl-MSCs blue) indicate the impossibility to discriminate these MSCs subpopulation at a transcriptional level. (d) Ingenuity pathway analysis was able to define enriched pathways implicated in embryonic morphogenesis and organ development in fetal MSCs compared to BM-MSCs. (e) Ingenuity pathway analysis using organ formation and osteoblast differentiation molecular network shows an enrichment in proosteogenic genes in fetal MSCs compared to BM-MSCs.

We first analyzed differences between membrane-derived and placenta-derived MSCs. As demonstrated by our PCA analysis, MSCs derived from membrane or placenta could not be differentiated based upon their transcriptomic profile (Figure 4(a)). We then analyzed the different subpopulations defined by CD90 and CD29 expression. They also displayed similar transcriptomic profile (Figures 4(b) and 4(c)).

When compared to BM-MSCs, 145 genes were significantly upregulated and 267 genes were downregulated in Mb-MSCs compared to BM-MSCs (Supplementary Table 1 and Supplementary Figure 5). Similarly, 154 genes were significantly upregulated (133 overlapping with Mb-MSCs upregulated genes) and 272 genes were downregulated (238 overlapping with Mb-MSCs downregulated genes) in the Pl-MSCs compared to BM-MSCs (Supplementary Table 2 and Supplementary Figure 5).

By ingenuity and David analysis, we were able to define several pathways and genes implicated in embryonic morphogenesis and organ development upregulated in the Pl and Mb-MSC compared to BM-MSC (Figure 4(d) and Supplementary Table 3). Several genes implicated in extracellular matrix organization, the skeletal system development and vasculature development were upregulated in the BM-MSC compared to Pl and Mb-MSC (Supplementary Table 4).

We then performed ingenuity pathway analysis building organ formation and osteoblast differentiation molecular networks. We found 14 genes upregulated in Pl/Mb MSC implicated in osteogenic differentiation in literature such as BMP, IGFBP4, IL6, HGF, and PTGS2 (Figure 4(e)).

### 3.8. Osteoactivin-Derived Osteoblast Differentiation

**Figure 5:**
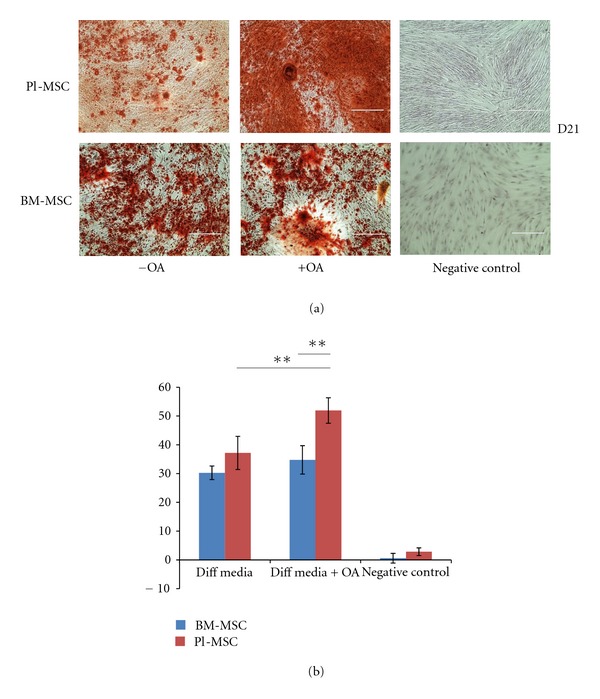
Osteoactivin trigger increased osteogenic differentiation in human fetal and bone marrow MSCs. (a) Illustration of increased osteogenic differentiation with OA compared to control (without OA) after 21 days in Mb-MSCs and BM-MSCs. (b) Quantification of osteogenic differentiation with or without Osteoactivin (**P < 0.05).

Amniotic-membrane-derived MSCs (Mb-MSCs) were used for this part of the study as they are similar to the MSCs derived from the placenta and more abundant. In our cell culture and differentiation settings, the Mb/Pl MSCs displayed no differences in their ability to differentiate toward osteoblasts compare to BM-MSCs. OA treatment increased the differentiation for both Mb-MSC and BM-MSC at days 14 and 21 as demonstrated by Alizarin red staining, with Mb-MSCs displaying significantly increased osteogenic differentiation (Figures 5(a) and 5(b)). We noticed that the addition of OA to the differentiation media accelerated osteogenic differentiation with positive Alizarin staining from day 7 for Mb-MSC and day 14 for BM-MSC (data not shown).

### 3.9. Phosphokinase Array Analysis of Differentiating Cells

**Figure 6:**
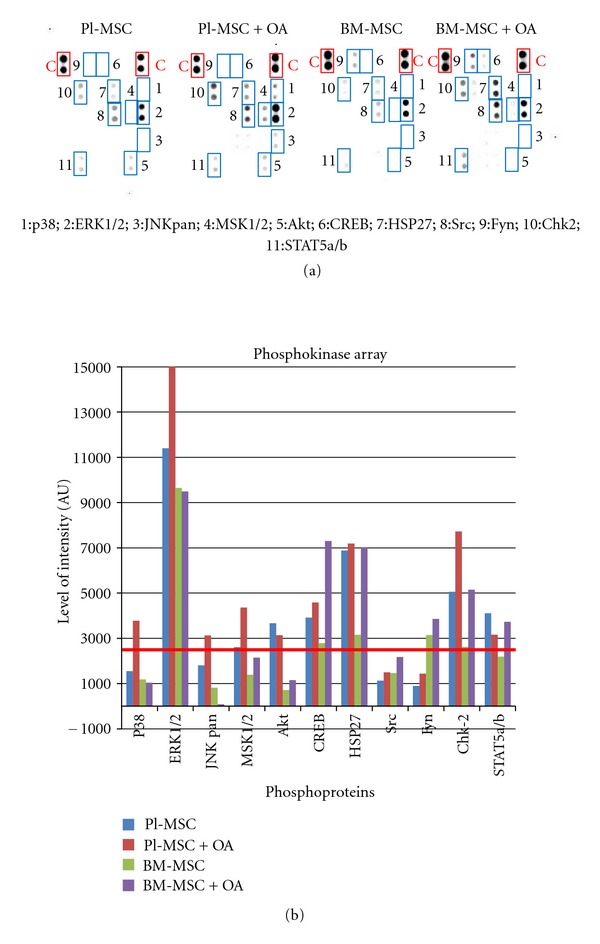
Phosphokinase array analysis 4 h after osteoactivin stimulation. After 4 h of OA stimulation, cells were harvested and protein extracts analyzed with the human Phosphokinase array from R&D system. (a) Phosphokinase profile in Pl-MSCs and BM-MSCs using the proteome profiler. (b) Quantification of phosphokinase optical density. Measurements were obtained with image J software (NIH). ERK2 is activated in placental MSCs when submitted to osteoactivin stimulation.

We analyzed the phosphorylation pattern of a range of phosphokinase after 4 h of OA stimulation in Pl-MSCs and BM-MSCs (Figures 6(a) and 6(b)). For both cell lines, there is a phosphorylation of Chk2 compatible with reduced proliferation during the differentiation process. While CREB is phosphorylated in BM-MSC, OA triggers ERK1/2 phosphorylation in Pl-MSCs. ERK activation was already previously described by Furochi et al. [40] as activated through OA. Those previous findings together with our datas lead us to stand for an OA activation role in osteogenic differentiation notably through ERK1/2 pathway activation in fetal-derived MSCs.

## 4. Discussion

MSCs are thought to have great therapeutic potential due to their capacity for self-renewal and multilineage differentiation [4, 41]. For example, they support hematopoiesis and enhance engraftment of hematopoietic stem cells after cotransplantation [3, 42]. Experimental and clinical data have demonstrated an immune-regulatory function of BM-derived MSCs that may contribute to the reduction of graft-versus-host disease following hematopoietic stem cell transplantation [43, 44]. Furthermore, even if clinical studies remain anecdotal, BM-MSCs have been reported to exert beneficial effects in the healing of a limited number of patients with bone nonunions [45–50]. MSCs initiate the fracture repair process leading to the formation of a cartilaginous template (callus) that is then replaced by new bone that fills the gap [6]. Limitation in MSC number and/or functions is hypothesized to play a critical role in the pathogenesis of post fracture nonunions.

Currently, the bone marrow is perceived as the major source of MSCs for cell therapy. However, aspiration of BM involves invasive procedures. The frequency, differentiation, and growth potential of BM-MSCs decrease significantly with age [51]. Thus, the search for alternative consistent sources of MSCs is of significant value. Indeed, when we consider therapeutic application, it will be mandatory to access cell banks displaying a large variety of HLA types. It has been reported that MSCs could be isolated from various tissues [11, 52]. Among these sources, placenta and membrane may be ideal sources due to their accessibility, painless donor procurement, promising sources for autologous cell therapy, and lower risk of viral contamination. The accessibility of these tissues will allow constituting clinically relevant banking program.

In this study, we have isolated MSCs from placenta and fetal membrane using very simple isolation technique with the same great purity yield (more than 95%) than initial FACS sorting methods [53]. Moreover, no difference was found between different MSCs subpopulation of placenta and membrane considering phenotypic characteristics, growth kinetic, markers expression, differentiation assays, and transcriptomic profile. This suggests the plasticity of certain MSC markers. We indeed illustrate that the surface markers used for MSCs cell sorting have limited interest in fetal mesenchymal stem cell tissues isolation. We demonstrate that the yield of MSCs retrieval is 6–8 fold superior in the fetal membranes than in placenta. In addition, others already demonstrated through cytogenetic analysis that placenta-derived MSCs maintained a normal karyotype for 30–40 passages *in vitro* [54]. Indeed, we demonstrate that the Mb/Pl MSCs retain even at high number of passages significantly better proliferation ability than BM-MSCs. We finally demonstrate that these fetal MSC share close transcriptomic profiles with BM-MSC.

Currently, bone morphogenetic protein-2 and -7 (BMP-2 and -7) are the only biologic modifiers that have received the United States Food and Drug Administration (US-FDA) approval for clinical applications in orthopedic surgery. The BMPs low biologic activity is demonstrated by the doses of tens of milligrams of commercial BMP-2- and -7-containing products, whereas BMPs concentrations *in vivo* are around several micrograms per kilogram of bone [55, 56]. BMP therapeutic doses in preclinical and clinical trials varied by factors up to 100 folds, demonstrating low consistency on bone repair [57].

Noteworthy, we have characterized the mesenchymal stem cells by the criteria used by the ISCT [33]. We would like to point a limitation emphasized by the plasticity of the phenotypic markers. First, the true stemness ablity (self-renewal) of our MSCs was not demonstrated and should be further documented in studies looking at clonality of the cell lines. Therefore, while they have a real ability to differentiate in different lineage, it is impossible to say if a single cell can indeed differentiate in different lineages. Moreover, the role of this cell types *in vivo* remains still not clearly define by lack of specific targeting of the mesenchymal stem cells.

We recently demonstrated that OA acts downstream of BMP-2, and our results indicate that OA may have similar osteoinductive effects to BMP-2 in mice.

We investigated the response of fetal MSCs to OA as compared to BM-MSCs. We demonstrate that OA can induce osteogenic differentiation in human MSCs. More interestingly, fetal-derived MSCs display better response to OA than BM-MSCs. We finally demonstrate that OA can also be used as a complement for osteogenic-induced differentiation with fetal MSCs. Finally, in accordance with the literature, we document that the induction of osteogenic differentiation following OA stimulation involved ERK1/2 pathway activation.

Considering that MSCs are way more abundant in membrane compared to placenta, we, therefore, stand that fetal membranes could be used to build MSCs banking program in order to meet clinical threshold in bone fracture reparation. Isolation of Mb-MSCs through selective culture in DMEM-low glucose supplemented with 20% serum and antibiotics seems to be the most efficient process. This process is very adapted for automation compatible with large cell banking programs.

Moreover, the increased capacity of response to osteogenic differentiation upon osteoactivin treatment prompts us to study the role of fetal membrane MSCs in experimental preclinical model of bone regeneration. Indeed, critical animal studies would be necessary to determine how MSCs are recruited and survive at the fracture site, their repair effectiveness, and the mechanisms through which they exert their actions.

## Supplementary Material

Figure 1: Workflow of MSCs isolation
Figure 2: Comprehensive analysis of MSCs markers on freshly isolated cells from the amniotic membrane.
Figure 3: Pl/Mb-MSCs constitute a very homogenous population after 4 passages.
Figure 4: Immunofluorescence staining of placenta and membrane derived MSCs.
Figure 5: Venn analysis of genes up and down regulated in Pl/Mb-MSCs as compared to BM-MSCs.
Table 1: Lists of genes differentially expressed between bone Marrow MSCs and fetal membrane MSCs.
Table 2: Lists of genes differentially expressed between bone Marrow MSCs and placenta MSCs.
Table 3: List of genes involved in developmental processes over-expressed in Mb-MSC compares to BM-MSC.
Table 4: List of genes involved in adhesion and matrix interaction over-expressed in BM-MSC compare to Mb-MSC.

## Figures and Tables

**Table 1:** Proportion of cell expressing MSCs specific markers. After 4 passages, every cell sorted subtype and bone marrow MSCs were stained for CD45, CD34, CD90, CD29, CD105, and CD73. 99% of the cells were CD34 and CD45 negative and more than 80% positive for the MSCs makers.

	CD90^+^	CD29^+^	CD105^+^	CD73^+^
Bone marrow	97.6%	90.9%	99.7%	98.7%
Membrane	87.6%	99.8%	96.7%	99.8%
Membrane CD29^+^ CD90^+^	79.3%	99.7%	96.4%	99.5%
Membrane CD29^+^ CD90^−^	91.8%	98%	98.1%	97.8%
Membrane CD29^−^ CD90^+^	85.2%	99.9%	98.5%	99.4%
Placenta	91.1%	99.8%	99.2%	99.5%
Placenta CD29^+^ CD90^+^	79.1%	99.9%	99.1%	99.9%
Placenta CD29^−^ CD90^+^	84.7%	99.6%	99.8%	99.5%
