# Supplementary material for: Comprehensive Characterization of Mesenchymal Stem Cells from Human Placenta and Fetal Membrane and Their Response to Osteoactivin Stimulation
Source: Stem Cells Int. 2012 Jun 4;2012:658356. doi: 10.1155/2012/658356 (PMC3373208; doi:10.1155/2012/658356)
Supplement: Supplementary file 1 — Figure 1: Workflow of MSCs isolation Figure 2: Comprehensive analysis of MSCs markers on freshly isolated cells from the amniotic membrane. Figure 3: Pl/Mb-MSCs constitute a very homogenous population after 4 passages. Figure 4: Immunofluorescence staining of placenta and membrane derived MSCs. Figure 5: Venn analysis of genes up and down regulated in Pl/Mb-MSCs as compared to BM-MSCs. Table 1: Lists of genes differentially expressed between bone Marrow MSCs and fetal membrane MSCs. Table 2: Lists of genes differentially expressed between bone Marrow MSCs and placenta MSCs. Table 3: List of genes involved in developmental processes over-expressed in Mb-MSC compares to BM-MSC. Table 4: List of genes involved in adhesion and matrix interaction over-expressed in BM-MSC compare to Mb-MSC. [file 658356.f1.pdf]

Supplementary Figure 1: Workflow of MSCs isolation

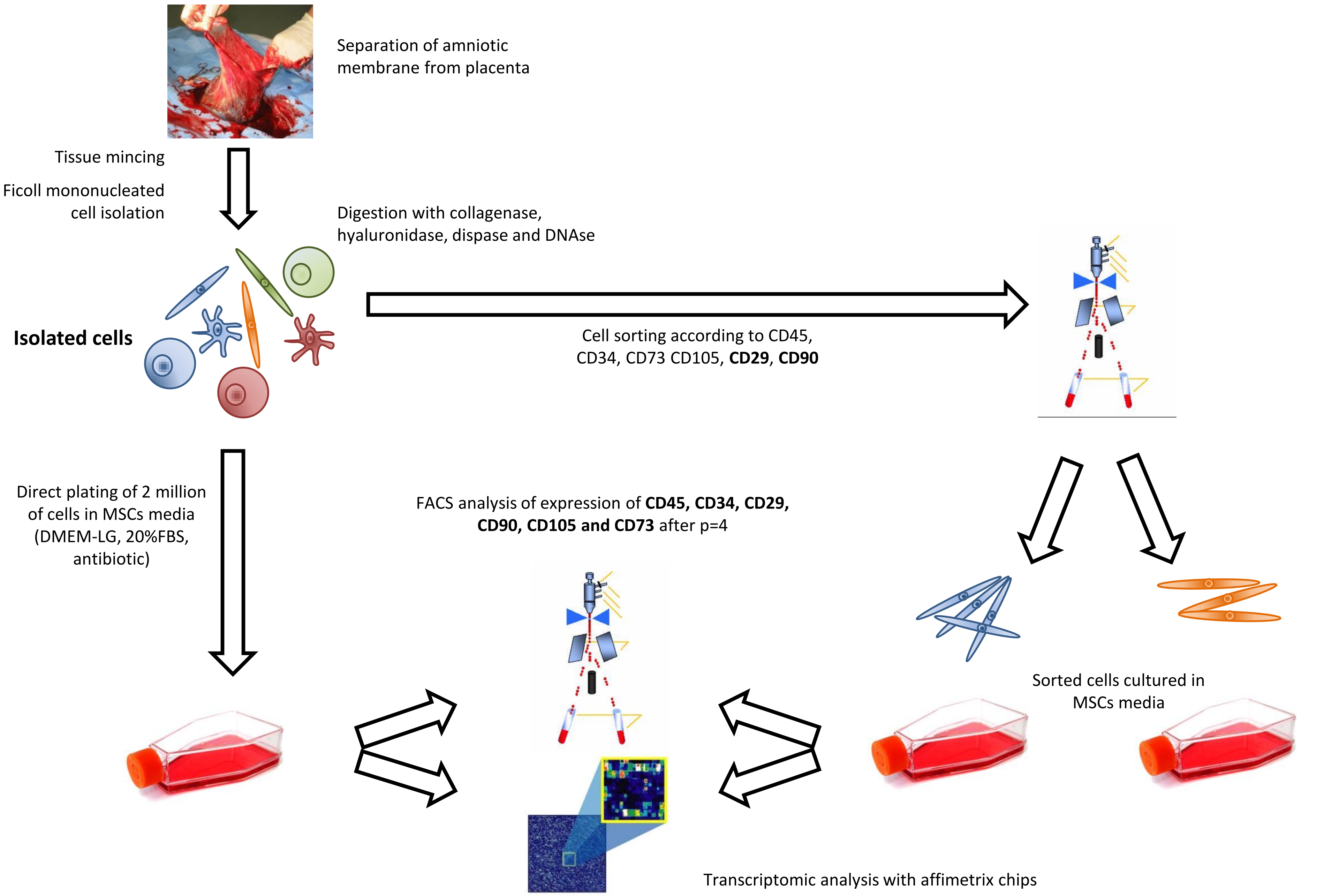

Supplementary Figure 2: Comprehensive analysis of MSCs markers on freshly isolated cells from the amniotic membrane.

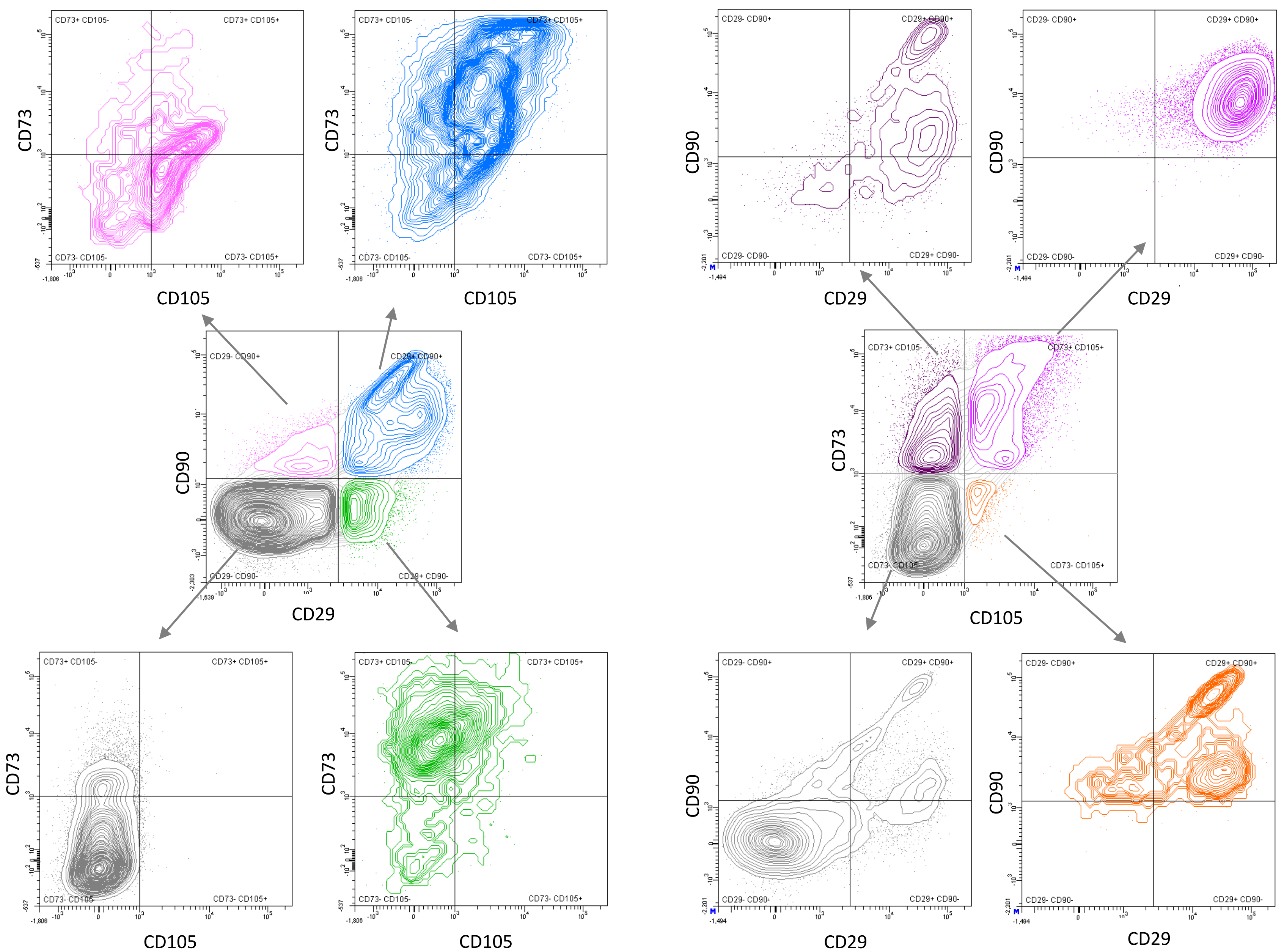

Supplementary Figure 3: Pl/Mb-MSCs constitute a very homogenous population after 4 passages

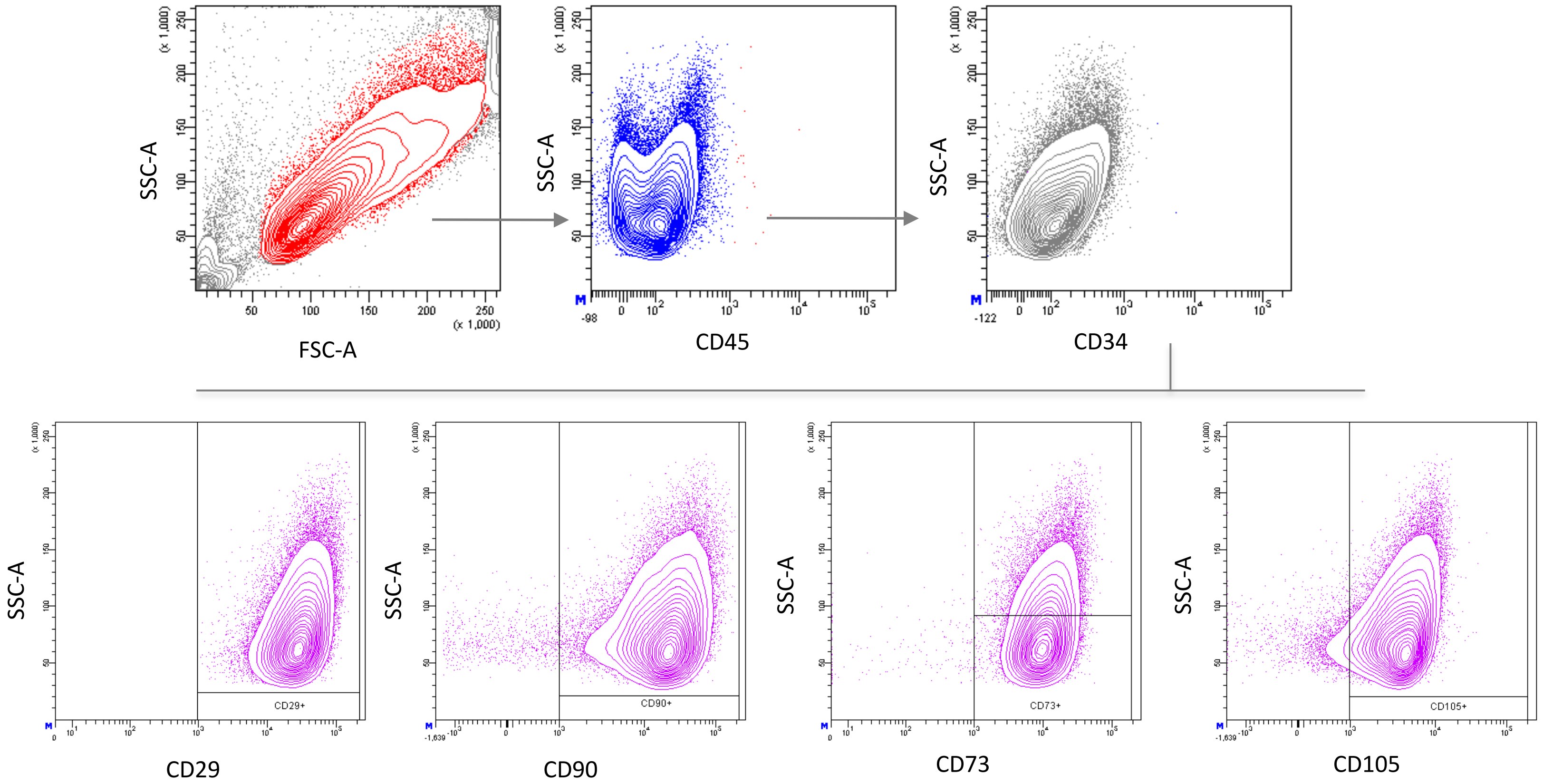

Supplementary Figure 4: Immunofluorescence staining of placenta and membrane derived MSCs.

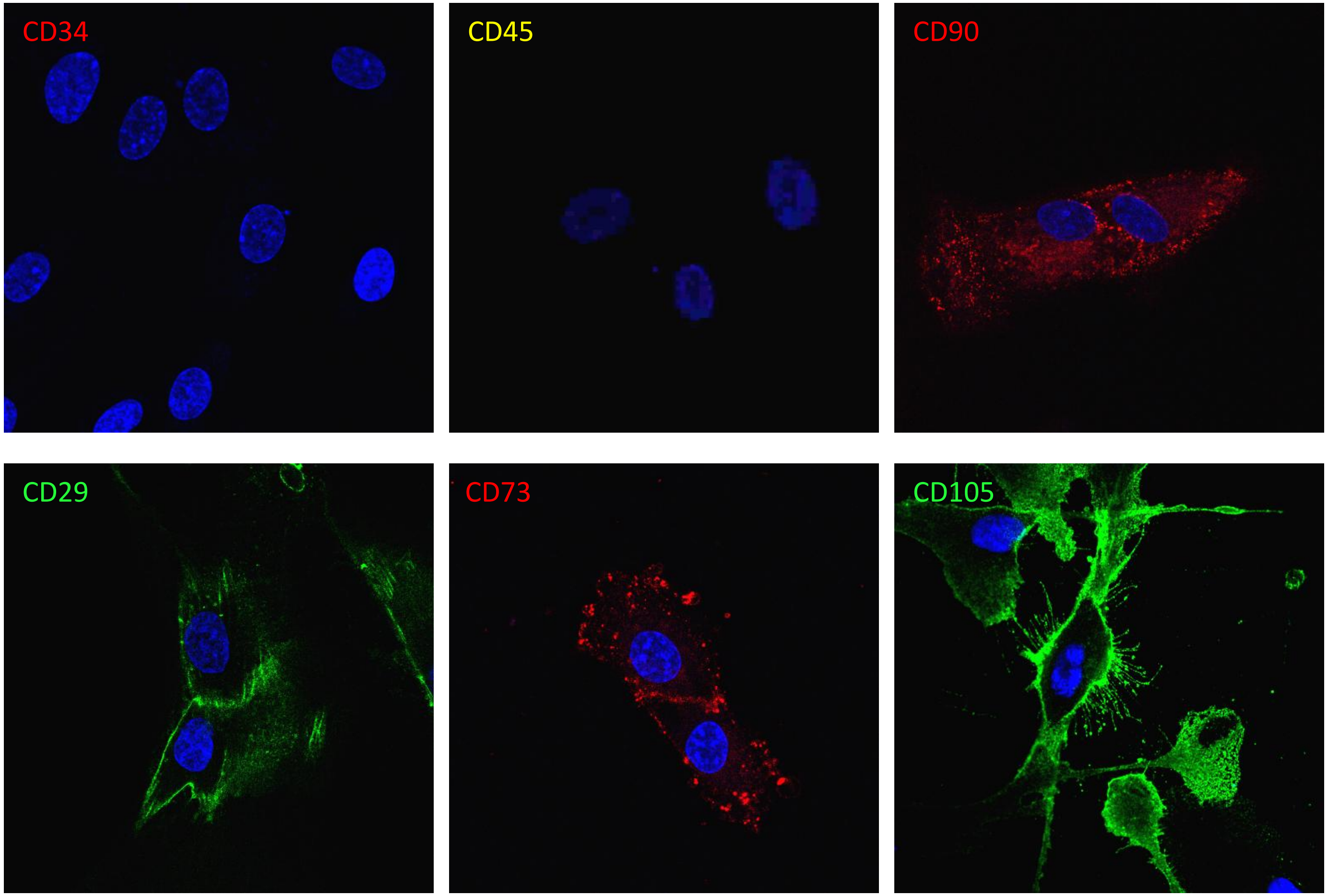

Supplementary Figure 5: Venn analysis of genes up and down regulated in Pl/Mb-MSCs as compared to BM-MSCs.

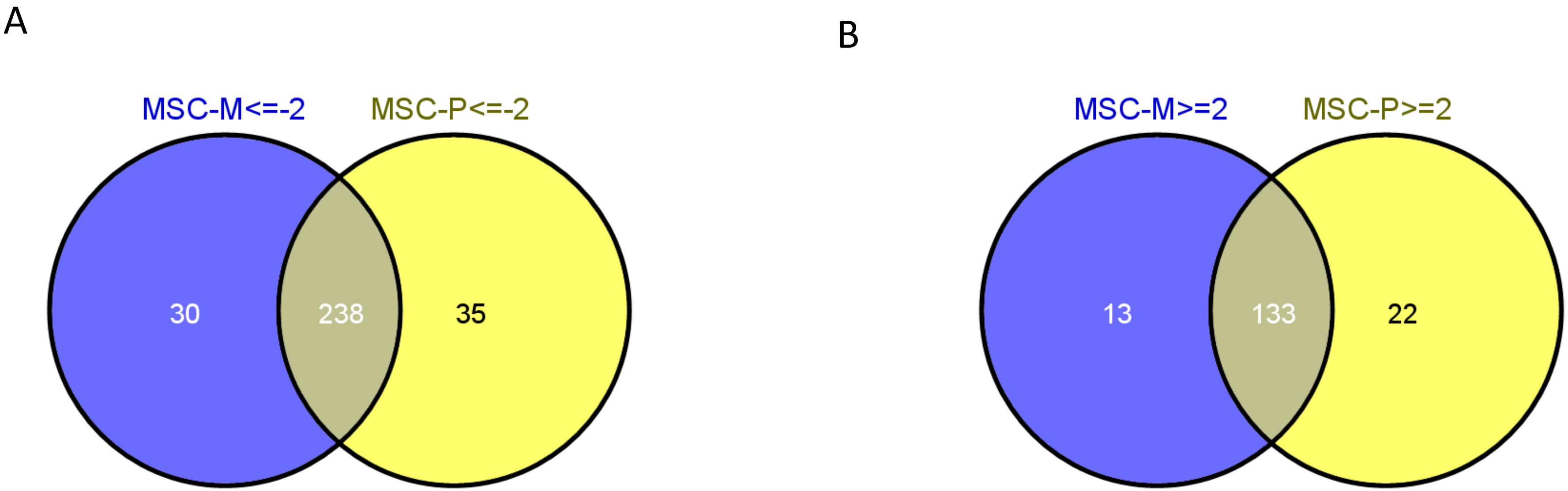

Supplementary Table 1: Lists of genes differentially expressed between bone Marrow MSCs and fetal membrane MSCs.

**Genes overexpressed in BMMSCs as compared to MbMSCs**			
**Gene name**	**Accession Nb**	**Fold increase**	**P value**
cytochrome P450, family 1, subfamily B, polypeptide 1	NM_000104	7.33	0.012897
elastin (supravalvular aortic stenosis, Williams-Beuren syndrome)	AA479278	6.33	0.010858
cytochrome P450, family 1, subfamily B, polypeptide 1	AU154504	6.11	0.010872
cytochrome P450, family 1, subfamily B, polypeptide 1	AU144855	5.93	0.00025
proenkephalin	NM_006211	5.86	0.000005
pentraxin-related gene, rapidly induced by IL-1 beta	NM_002852	5.56	0.005349
potassium channel tetramerisation domain containing 12	AI718937	5.55	0.002833
EGF-containing fibulin-like extracellular matrix protein 1	AI826799	5.51	0.004207
tissue inhibitor of metalloproteinase 3 (Sorsby fundus dystrophy, pseudoinflammatory)	NM_000362	5.47	0.000003
iroquois homeobox protein 3	AI681917	5.26	0.004546
tissue inhibitor of metalloproteinase 3 (Sorsby fundus dystrophy, pseudoinflammatory)	U67195	4.84	0.000004
paired-like homeodomain transcription factor 2	NM_000325	4.83	0.003789
phosphatidic acid phosphatase type 2B	AV725664	4.71	0.000173
tissue inhibitor of metalloproteinase 3 (Sorsby fundus dystrophy, pseudoinflammatory)	BF347089	4.7	0.000195
hyaluronan and proteoglycan link protein 1	U43328	4.69	0.000637
UDP-N-acetyl-alpha-D-galactosamine:polypeptide N-acetylgalactosaminyltransferase 5 (GalNAc-T5)	AK022198	4.68	0.000282
Hyaluronan and proteoglycan link protein 1	AU144114	4.67	0.001581
filaggrin	AL356504	4.66	0.000892
fibronectin type III domain containing 1	AI345957	4.66	0.002226
prostaglandin I2 (prostacyclin) synthase /// prostaglandin I2 (prostacyclin) synthase	NM_000961	4.57	0.02255
EGF-containing fibulin-like extracellular matrix protein 1	NM_004105	4.53	0.011438
ribosomal protein S4, Y-linked 1	NM_001008	4.5	0.000034
prostaglandin-endoperoxide synthase 2 (prostaglandin G/H synthase and cyclooxygenase)	NM_000963	4.49	0.000164
ankyrin 3, node of Ranvier (ankyrin G)	NM_020987	4.48	0.000069

potassium channel tetramerisation domain containing 12 /// potassium channel tetramerisation domain containing 12	AA551075	4.34	0.000002
tissue inhibitor of metalloproteinase 3 (Sorsby fundus dystrophy, pseudoinflammatory)	AW338933	4.23	0.004671
keratin associated protein 1-1 /// keratin associated protein 1-1	NM_030967	4.21	0.001068
crystallin, alpha B	AF007162	4.18	0.000058
aldehyde dehydrogenase 1 family, member A3	NM_000693	4.15	0.000002
paired related homeobox 2	NM_016307	4.13	0.000009
importin 9	AI659533	3.97	0.001591
ABI gene family, member 3 (NESH) binding protein	AB056106	3.94	0.001912
integrin, beta-like 1 (with EGF-like repeat domains)	NM_004791	3.91	0.001888
ectonucleotide pyrophosphatase/phosphodiesterase 2 (autotaxin)	L35594	3.88	0.001338
microfibrillar associated protein 5	AW665892	3.86	0.005145
dapper homolog 1, antagonist of beta-catenin (xenopus)	NM_016651	3.85	0.00044
hypothetical protein LOC283824	AI693140	3.77	0.006055
transforming growth factor, beta-induced, 68kDa	NM_000358	3.74	0.004246
hypothetical protein DKFZp434L142	AF260333	3.74	0.010262
gb:AL359052.1 /DB_XREF=gi:8518175 /FEA=mRNA /CNT=13 /TID=Hs.311054.0 /TIER=ConsEnd /STK=5 /UG=Hs.311054 /DEF=Homo sapiens mRNA full length insert cDNA clone EUROIMAGE 1968422. /PROD=ITGBL1, integrin beta-like 1	AL359052	3.72	0.002959
CDNA FLJ31059 fis, clone HSYRA2000832	AA418028	3.72	0.012902
phosphatidic acid phosphatase type 2B	AA628586	3.71	0.000009
UDP-N-acetyl-alpha-D-galactosamine:polypeptide N-acetylgalactosaminyltransferase 1 (GalNAc-T1)	BC038440	3.71	0.00319
solute carrier family 14 (urea transporter), member 1 (Kidd blood group)	NM_015865	3.66	0.000542
receptor tyrosine kinase-like orphan receptor 1	NM_005012	3.65	0.000712
CDNA: FLJ23131 fis, clone LNG08502	AK026784	3.65	0.00001
supervillin	NM_003174	3.64	0.002608
prostaglandin E synthase	NM_004878	3.64	0.003281
UDP-N-acetyl-alpha-D-galactosamine:polypeptide N-acetylgalactosaminyltransferase 1 (GalNAc-T1)	AV692127	3.63	0.004216
vascular cell adhesion molecule 1	NM_001078	3.59	0.006421
lumican	NM_002345	3.58	0.013693
phosphatidic acid phosphatase type 2B	AB000889	3.58	0.003956
decorin	AF138302	3.58	0.000003
Homo sapiens, clone IMAGE:4794726, mRNA	BF513121	3.58	0.022143
GATA binding protein 6	D87811	3.57	0.001547

Integrin, beta-like 1 (with EGF-like repeat domains)	AI753143	3.57	0.000531
cyclin-dependent kinase inhibitor 2B (p15, inhibits CDK4)	AW444761	3.56	0.000531
decorin	AF138300	3.5	0.000015
endothelial cell-specific molecule 1	NM_007036	3.5	0.030103
hyaluronan and proteoglycan link protein 1	NM_001884	3.48	0.00047
CDNA clone IMAGE:4152983, partial cds	BF344237	3.48	0.000085
integrin, alpha 11	AF109681	3.48	0.000035
UDP-N-acetyl-alpha-D-galactosamine:polypeptide N-acetylgalactosaminyltransferase 1 (GalNAc-T1)	NM_020474	3.47	0.019985
major histocompatibility complex, class II, DP alpha 1	M27487	3.47	0.000351
Fibronectin 1	AI333596	3.42	0.000247
DEAD (Asp-Glu-Ala-Asp) box polypeptide 3, Y-linked	NM_004660	3.41	0.003436
chitinase 3-like 1 (cartilage glycoprotein-39)	M80927	3.41	0.014534
decorin	AF138303	3.41	0.000009
UDP-N-acetyl-alpha-D-galactosamine:polypeptide N-acetylgalactosaminyltransferase 1 (GalNAc-T1)	U41514	3.38	0.003091
serine (or cysteine) proteinase inhibitor, clade E (nexin, plasminogen activator inhibitor type 1), member 2	AL541302	3.38	0.025419
Solute carrier family 14 (urea transporter), member 1 (Kidd blood group)	BE673587	3.37	0.000284
hepatocyte growth factor (hepapoietin A; scatter factor)	X16323	3.36	0.013948
tumor protein D52-like 1	NM_003287	3.33	0.065116
pregnancy-associated plasma protein A, pappalysin 1	BF107618	3.33	0.001763
prostaglandin E synthase	AF010316	3.32	0.001619
gb:BF002195 /DB_XREF=gi:10702470 /DB_XREF=7h23h07.x1 /CLONE=IMAGE:3316861 /FEA=EST /CNT=8 /TID=Hs.131624.0 /TIER=ConsEnd /STK=6 /UG=Hs.131624 /UG_TITLE=ESTs	BF002195	3.31	0.000009
lysyl oxidase	L16895	3.27	0.012891
EGF, latrophilin and seven transmembrane domain containing 1	NM_022159	3.27	0.008722
ankylosis, progressive homolog (mouse)	AA854943	3.27	0.000123
interleukin 6 (interferon, beta 2)	NM_000600	3.2	0.014285
microfibrillar associated protein 5	AW665892	3.2	0.000561
microfibrillar associated protein 5	U37283	3.17	0.000114
hypothetical protein FLJ14054	NM_024563	3.17	0.011334
biglycan /// serologically defined colon cancer antigen 33	AA845258	3.14	0.002314
Hyaluronan synthase 2	AI374739	3.14	0.033309
adducin 3 (gamma)	NM_019903	3.12	0.001478
chemokine (C-X-C motif) ligand 12 (stromal cell-derived factor 1)	U19495	3.11	0.020188

KIAA0746 protein	AB018289	3.11	0.012906
pregnancy-associated plasma protein A, pappalysin 1	AI110886	3.11	0.004259
gb:AW044286 /DB_XREF=gi:5904815 /DB_XREF=wv67c06.x1 /CLONE=IMAGE:2534602 /FEA=EST /CNT=19 /TID=Hs.174134.2 /TIER=Stack /STK=18 /UG=Hs.174134 /LL=51204 /UG_GENE=LOC51204 /UG_TITLE=clone HQ0477 PRO0477p	AW044286	3.1	0.002618
KIAA1199	AB033025	3.09	0.000161
ankyrin repeat domain 1 (cardiac muscle)	NM_014391	3.08	0.000087
Ubiquitin specific protease 9, Y-linked (fat facets-like, Drosophila)	AV681765	3.08	0.002173
CD74 antigen (invariant polypeptide of major histocompatibility complex, class II antigen-associated)	K01144	3.07	0.006218
biglycan	BC002416	3.06	0.000576
Forkhead box C2 (MFH-1, mesenchyme forkhead 1)	BF446723	3.05	0.000519
Hyaluronan and proteoglycan link protein 1	AI760252	3.04	0.000025
early B-cell factor 3	AL354950	3.03	0.000191
similar to expressed sequence AI593442	H24398	3	0.002891
tumor necrosis factor, alpha-induced protein 3	NM_006290	2.99	0.000184
adducin 3 (gamma)	AI818488	2.99	0.000002
gb:AW964972 /DB_XREF=gi:8154808 /DB_XREF=EST377045 /FEA=EST /CNT=66 /TID=Hs.48516.1 /TIER=Stack /STK=50 /UG=Hs.48516 /UG_TITLE=ESTs	AW964972	2.99	0.001636
insulin-like growth factor binding protein 3	BF340228	2.98	0.000097
DEP domain containing 6	NM_022783	2.98	0.008254
v-maf musculoaponeurotic fibrosarcoma oncogene homolog (avian)	AF055376	2.96	0.000236
insulin-like growth factor binding protein 3	M31159	2.95	0.000168
pregnancy-associated plasma protein A, pappalysin 1	BF107618	2.95	0.000422
decorin	AI281593	2.94	0.002426
placental growth factor, vascular endothelial growth factor-related protein	BC001422	2.94	0.001983
dehydrogenase/reductase (SDR family) member 3	NM_004753	2.93	0.044484
chitinase 3-like 1 (cartilage glycoprotein-39)	M80927	2.93	0.00636
Kruppel-like factor 4 (gut)	BF514079	2.93	0.000177
Ifapsoriasin	BC029685	2.93	0.006046
adducin 3 (gamma)	BE545756	2.92	0.007616
adducin 3 (gamma)	AI763123	2.91	0.002057
hephaestin	NM_014799	2.91	0.000072
cartilage oligomeric matrix protein	NM_000095	2.91	0.000958
chromosome 7 open reading frame 10	NM_024728	2.91	0.000619

cytoglobin	AL513673	2.89	0.002445
hemicentin	BF446673	2.87	0.002542
fibroblast growth factor 7 (keratinocyte growth factor)	NM_002009	2.86	0.011871
pregnancy-associated plasma protein A, pappalysin 1	AA148534	2.84	0.000014
chondroitin sulfate proteoglycan 2 (versican)	NM_004385	2.84	0.048368
serine/threonine kinase 38 like	AW779556	2.84	0.000755
chondroitin sulfate proteoglycan 2 (versican)	BF218922	2.84	0.051535
hypothetical protein FLJ38725	AI659800	2.84	0.008957
cell cycle progression 1	NM_004748	2.79	0.031828
kelch-like 13 (Drosophila)	AB037730	2.79	0.003817
complement factor H /// complement factor H-related 1	X56210	2.78	0.009071
hypothetical protein FLJ14834	AW084730	2.78	0.000052
ATP-binding cassette, sub-family A (ABC1), member 8	NM_007168	2.77	0.009808
latrophilin 2	NM_012302	2.77	0.002049
ankyrin repeat domain 29	AI307802	2.77	0.003593
homeo box C10	NM_017409	2.76	0.000002
core 1 synthase, glycoprotein-N-acetylgalactosamine 3-beta-galactosyltransferase, 1	NM_020156	2.76	0.005101
insulin-like growth factor binding protein 2, 36kDa	NM_000597	2.74	0.005157
SMAD, mothers against DPP homolog 6 (Drosophila)	NM_005585	2.74	0.002854
periostin, osteoblast specific factor	AY140646	2.73	0.003773
stanniocalcin 2	AI435828	2.71	0.000868
aldo-keto reductase family 1, member C3 (3-alpha hydroxysteroid dehydrogenase, type II)	AB018580	2.71	0.107687
heme oxygenase (decycling) 1	NM_002133	2.7	0.000176
transforming growth factor, beta receptor I (activin A receptor type II-like kinase, 53kDa)	AA604375	2.7	0.01479
hypothetical gene supported by BC009385	AL520272	2.7	0.000024
collagen triple helix repeat containing 1	AA584310	2.69	0.000252
lysyl oxidase	NM_002317	2.68	0.004286
KIAA0367	AB002365	2.67	0.000041
jagged 1 (Alagille syndrome)	U73936	2.63	0.009975
Full-length cDNA clone CS0DD009YB17 of Neuroblastoma Cot 50-normalized of Homo sapiens (human)	AK000776	2.63	0.012529
CDNA FLJ44429 fis, clone UTERU2015653	AI088063	2.62	0.048991
adiponutrin	AK025665	2.62	0.007312
NACHT, leucine rich repeat and PYD (pyrin domain) containing 1	NM_021730	2.6	0.010521

hyaluronan synthase 2	NM_005328	2.59	0.022251
jagged 1 (Alagille syndrome)	U77914	2.59	0.00968
Full length insert cDNA clone ZD77F06	AI655611	2.59	0.008924
SMAD, mothers against DPP homolog 6 (Drosophila)	AI193899	2.58	0.00196
pregnancy-associated plasma protein A, pappalysin 1	BG434272	2.55	0.008046
Full-length cDNA clone CS0DI014YH21 of Placenta Cot 25-normalized of Homo sapiens (human)	BF058422	2.54	0.001347
zinc finger protein 503	AA603467	2.54	0.001422
putative small membrane protein NID67	AF313413	2.53	0.005377
Jagged 1 (Alagille syndrome)	AI457817	2.53	0.000606
chondroitin sulfate proteoglycan 2 (versican) /// chondroitin sulfate proteoglycan 2 (versican)	R94644	2.52	0.039853
Core 1 synthase, glycoprotein-N-acetylgalactosamine 3-beta-galactosyltransferase, 1	AI672489	2.52	0.002588
Collagen, type VIII, alpha 1	AL359062	2.52	0.000237
fibronectin 1	X02761	2.51	0.00053
chondroitin sulfate proteoglycan 2 (versican)	BF590263	2.5	0.058619
gb:AA954994 /DB_XREF=gi:3118689 /DB_XREF=op24f03.s1 /CLONE=IMAGE:1577789 /FEA=EST /CNT=15 /TID=Hs.237396.1 /TIER=ConsEnd /STK=1 /UG=Hs.237396 /UG_TITLE=ESTs	AA954994	2.49	0.000628
fibronectin 1	AF130095	2.48	0.00132
EGF-like repeats and discoidin I-like domains 3	AA053711	2.48	0.028579
stanniocalcin 2	BC000658	2.47	0.00262
Similar to circumsporozoite protein precursor - Plasmodium brasilianum	H10408	2.47	0.005108
insulin-like growth factor binding protein 4	NM_001552	2.46	0.020264
fibronectin 1 /// fibronectin 1	BC005858	2.46	0.000361
major histocompatibility complex, class II, DR beta 1	AJ297586	2.46	0
transmembrane, prostate androgen induced RNA	AL035541	2.46	0.033789
CDNA FLJ26539 fis, clone KDN09310	AW025579	2.45	0.006846
heat shock 27kDa protein family, member 7 (cardiovascular)	NM_014424	2.45	0.000191
procollagen C-endopeptidase enhancer 2	NM_013363	2.45	0.000153
periostin, osteoblast specific factor	AY140646	2.44	0.020504
fibronectin 1	AK026737	2.43	0.00084
phosphoglucomutase 1	NM_002633	2.42	0.000453
phospholipase A2, group IVA (cytosolic, calcium-dependent)	M68874	2.42	0.010441
protein phosphatase 1, regulatory (inhibitor) subunit 3C	N26005	2.41	0.000098
fibronectin 1	AJ276395	2.39	0.003929
Transcribed locus	AI761573	2.39	0.039653

vascular endothelial growth factor	AF022375	2.38	0.000351
UDP-N-acetyl-alpha-D-galactosamine:polypeptide N-acetylgalactosaminyltransferase 5 (GalNAc-T5)	AI633503	2.38	0.00005
connective tissue growth factor	M92934	2.36	0.015837
fibronectin 1	AJ276395	2.36	0.000425
BMP and activin membrane-bound inhibitor	NM_012342	2.35	0.000426
zinc finger protein 423	AW149417	2.34	0.002103
sine oculis homeobox homolog 1 (Drosophila)	NM_005982	2.32	0.012124
hyaluronan synthase 1	NM_001523	2.32	0.003651
MRNA; cDNA DKFZp313C0240 (from clone DKFZp313C0240)	H16409	2.32	0.027062
fibroblast growth factor 7 (keratinocyte growth factor) /// hypothetical protein FLJ30435	AF523265	2.32	0.038532
aldo-keto reductase family 1, member C2 (dihydrodiol dehydrogenase 2; bile acid binding protein; 3-alpha hydroxysteroid dehydrogenase, type III) /// aldo-keto reductase family 1, member C2 (dihydrodiol dehydrogenase 2; bile acid binding protein; 3-alpha hydroxysteroid dehydrogenase, type III)	M33376	2.31	0.005436
natriuretic peptide receptor C/guanylate cyclase C (atrionatriuretic peptide receptor C)	AI628360	2.31	0.005662
lysyl oxidase-like 3	AI917716	2.3	0.005012
hypothetical LOC389048	BG285837	2.3	0.018044
cadherin 6, type 2, K-cadherin (fetal kidney)	AU151483	2.29	0.000212
Doublecortin and CaM kinase-like 1	AI129626	2.29	0.00379
cytokine receptor-like factor 1	NM_004750	2.28	0.002113
FYN oncogene related to SRC, FGR, YES	M14333	2.28	0.004506
phosphodiesterase 4D interacting protein (myomegalin)	AB042557	2.28	0.001622
glutaminase	NM_014905	2.27	0.003222
aggrecan 1 (chondroitin sulfate proteoglycan 1, large aggregating proteoglycan, antigen identified by monoclonal antibody A0122)	NM_013227	2.27	0.000454
chromosome 5 open reading frame 13	NM_004772	2.26	0.005727
major histocompatibility complex, class II, DR beta 1 /// major histocompatibility complex, class II, DR beta 1	U65585	2.26	0.000136
periostin, osteoblast specific factor	D13665	2.26	0.028548
WAP four-disulfide core domain 1	NM_021197	2.26	0.001431
Ras association (RalGDS/AF-6) and pleckstrin homology domains 1	AA194149	2.26	0.004622
glutathione peroxidase 7	AA406605	2.25	0.001144
ependymin related protein 1 (zebrafish)	BC000686	2.25	0.000213
netrin 4	AF278532	2.25	0.036148
docking protein 1, 62kDa (downstream of tyrosine kinase 1)	AF035299	2.24	0.000392

CD24 antigen (small cell lung carcinoma cluster 4 antigen)	AA761181	2.23	0.059119
Hypothetical gene supported by AF275804	AA954994	2.23	0.000604
bone morphogenetic protein 1	NM_001199	2.22	0.00161
LIM domain only 2 (rhombotin-like 1)	NM_005574	2.21	0.001691
neurotrimin	AW085558	2.21	0.016539
Glypican 6	AI651255	2.2	0.023473
Transcribed locus	BF513800	2.2	0.013426
glutaminase	AF097493	2.19	0.01056
keratin 23 (histone deacetylase inducible)	NM_015515	2.19	0.007531
cathepsin D (lysosomal aspartyl protease)	AV706254	2.19	0.002301
gb:AB020690.1 /DB_XREF=gi:4240254 /GEN=KIAA0883 /FEA=FLmRNA /CNT=85 /TID=Hs.7782.0 /TIER=FL+Stack /STK=20 /UG=Hs.7782 /LL=10687 /DEF=Homo sapiens mRNA for KIAA0883 protein, complete cds. /PROD=KIAA0883 protein /FL=gb:NM_007257.1 gb:AB020690.1 gb:AF286487.1	AB020690	2.18	0.002667
aldo-keto reductase family 1, member C2 (dihydrodiol dehydrogenase 2; bile acid binding protein; 3-alpha hydroxysteroid dehydrogenase, type III)	U05598	2.18	0.089865
phosphodiesterase 7B	AI638433	2.18	0.000407
cysteine-rich protein 1 (intestinal)	NM_001311	2.17	0.001278
cadherin 11, type 2, OB-cadherin (osteoblast)	D21254	2.17	0.00179
KIAA1671 protein	AB051458	2.17	0.003885
family with sequence similarity 43, member A	AW264102	2.17	0.009784
bone morphogenetic protein 1	NM_006132	2.15	0.003523
KIAA1644 protein	BF516072	2.15	0.000625
CD24 antigen (small cell lung carcinoma cluster 4 antigen)	L33930	2.15	0.053754
tissue inhibitor of metalloproteinase 1 (erythroid potentiating activity, collagenase inhibitor)	NM_003254	2.14	0.000197
growth arrest-specific 1	NM_002048	2.14	0.016595
gap junction protein, alpha 1, 43kDa (connexin 43)	NM_000165	2.12	0.020671
ectonucleotide pyrophosphatase/phosphodiesterase 2 (autotaxin)	D45421	2.12	0.00054
gb:BC042886.1 /DB_XREF=gi:27503667 /TID=Hs2.127876.1 /CNT=3 /FEA=mRNA /TIER=ConsEnd /STK=1 /UG=Hs.127876 /UG_TITLE=Homo sapiens, clone IMAGE:5273182, mRNA /DEF=Homo sapiens, clone IMAGE:5273182, mRNA.	BC042886	2.12	0.010966
Methyltransferase 5 domain containing 1	AK091261	2.12	0.035293
Homo sapiens, Similar to HERV-H LTR-associating 3, clone IMAGE:4830680, mRNA	BC031956	2.12	0.008667
4-aminobutyrate aminotransferase	AF237813	2.11	0.01767
interferon-induced protein 44	NM_006417	2.11	0.003538
leukemia inhibitory factor (cholinergic differentiation factor)	NM_002309	2.1	0.006087

chromosome 10 open reading frame 54	BE271644	2.1	0.008587
leptin receptor	U50748	2.08	0.002045
zinc finger protein 324	NM_014347	2.07	0.002588
aryl hydrocarbon receptor	NM_001621	2.06	0.000804
midline 1 (Opitz/BBB syndrome)	NM_000381	2.06	0.000642
KIAA1102 protein	AK026815	2.05	0.001853
frizzled homolog 8 (Drosophila)	AW340311	2.05	0.000275
Transcribed locus	BG236273	2.05	0.000793
similar to AVLV472	BC021680	2.05	0.003848
EPH receptor A3	AF213459	2.04	0.000059
twist homolog 1 (acrocephalosyndactyly 3; Saethre-Chotzen syndrome) (Drosophila)	X99268	2.04	0.000443
frizzled homolog 8 (Drosophila) /// frizzled homolog 8 (Drosophila)	AB043703	2.04	0.006428
ankyrin 2, neuronal	BF726212	2.03	0.007184
frizzled homolog 1 (Drosophila)	NM_003505	2.03	0.000091
ankylosis, progressive homolog (mouse)	T99215	2.03	0.000498
microtubule-associated protein 1B	AA554833	2.03	0.001507
ectodermal-neural cortex (with BTB-like domain)	AF010314	2.02	0.001284
collagen, type VIII, alpha 2	AI806793	2.02	0.014233
nicotinamide N-methyltransferase	NM_006169	2.01	0.010349
serine (or cysteine) proteinase inhibitor, clade F (alpha-2 antiplasmin, pigment epithelium derived factor), member 1	NM_002615	2.01	0.003976
Adipocyte-specific adhesion molecule	BG112263	2.01	0.00072
leucine-rich repeat-containing G protein-coupled receptor 4	NM_018490	2	0.010269
LOC440156	AL530703	2	0.000325
Serine (or cysteine) proteinase inhibitor, clade E (nexin, plasminogen activator inhibitor type 1), member 2	AA703280	2	0.006496

Genes overexpressed in Mb-MSCs as compared to BM-MSCs			
**Gene name**	**Accession Nb**	**Fold increase**	**P value**
chromosome 10 open reading frame 58	BF512388	2	0.020095
putative homeodomain transcription factor 2	AL136883	2.01	0.017603
tetraspanin 5 /// tetraspanin 5	AF065389	2.01	0.000076
Dedicator of cytokinesis 2	AI991459	2.02	0.017508
phorbol-12-myristate-13-acetate-induced protein 1	AI857639	2.03	0.011667
nucleoredoxin	NM_017821	2.03	0.002391
guanylate binding protein 3	AL136680	2.03	0.033813
gb:AI963304 /DB_XREF=gi:5756017 /DB_XREF=wt61d01.x1 /CLONE=IMAGE:2511937 /FEA=EST /CNT=18 /TID=Hs.110373.0 /TIER=Stack /STK=13 /UG=Hs.110373 /UG_TITLE=ESTs	AI963304	2.03	0.016889
tribbles homolog 1 (Drosophila)	NM_025195	2.04	0.009209
leucine rich repeat containing 17	NM_005824	2.04	0.00018
nucleolar and spindle associated protein 1	NM_018454	2.04	0.000496
Growth arrest-specific 2 like 3	AI860012	2.04	0.010473
hypothetical LOC387763	AW276078	2.06	0.002767
kinesin family member 2C	U63743	2.1	0.000541
PDZ and LIM domain 1 (elfin)	BC000915	2.11	0.013929
calponin 1, basic, smooth muscle	NM_001299	2.13	0.003582
serine/threonine kinase 6	NM_003600	2.13	0.001585
MCM10 minichromosome maintenance deficient 10 (S. cerevisiae)	AL136840	2.13	0.025608
Full-length cDNA clone CS0DK010YC18 of HeLa cells Cot 25-normalized of Homo sapiens (human)	AI521166	2.13	0.065313
agrin	AK021586	2.14	0.047548
churchill domain containing 1	BE568660	2.14	0.023507
transcription factor 19 (SC1)	BC002493	2.15	0.011675
aminoacylase 1-like 2	AI654133	2.15	0.020177
stanniocalcin 1	AI300520	2.16	0.000327
cell division cycle associated 7 /// cell division cycle associated 7	AY029179	2.16	0.00483
apolipoprotein B mRNA editing enzyme, catalytic polypeptide-like 3B	NM_004900	2.17	0.001528
collagen, type VII, alpha 1 (epidermolysis bullosa, dystrophic, dominant and recessive)	NM_000094	2.18	0.005335
stanniocalcin 1	NM_003155	2.18	0.003147
ARG99 protein	AU151239	2.18	0.000208

chromosome 20 open reading frame 129	BC001068	2.2	0.008017
Hypothetical protein FLJ13273	AA740875	2.2	0.035591
hairy and enhancer of split 1, (Drosophila)	BE973687	2.22	0.003364
associated molecule with the SH3 domain of STAM (AMSH) like protein	AI638611	2.22	0.005625
E2F transcription factor 7	AI341146	2.22	0.001256
Stanniocalcin 1	AW003173	2.22	0.001695
hypothetical protein LOC146909	AA292789	2.23	0.000239
sal-like 1 (Drosophila)	AU152837	2.23	0.041534
FERM domain containing 4A	AW515645	2.24	0.004551
KIAA0186 gene product	NM_021067	2.25	0.002412
thyrotropin-releasing hormone degrading ectoenzyme	NM_013381	2.25	0.001138
hypothetical protein MGC11324 /// hypothetical protein MGC11324	BC006236	2.26	0.008964
L1 cell adhesion molecule	AI653981	2.27	0.014375
solute carrier family 7 (cationic amino acid transporter, y+ system), member 2	AA876372	2.27	0.003413
elastin microfibril interfacer 2 /// elastin microfibril interfacer 2	AF270513	2.29	0.001206
S100 calcium binding protein A4 (calcium protein, calvasculin, metastasin, murine placental homolog)	NM_002961	2.3	0.030394
glutathione S-transferase theta 1	NM_000853	2.3	0.009944
ribonucleotide reductase M2 polypeptide	BC001886	2.3	0.000397
Nuclear receptor subfamily 2, group F, member 1	AI951185	2.31	0.016112
insulin receptor substrate 2	BF700086	2.32	0.002012
aldehyde oxidase 1	NM_001159	2.33	0.003584
agrin	AW008051	2.34	0.020921
hypothetical protein MGC45871	AI961778	2.34	0.000403
fatty acid binding protein 4, adipocyte	NM_001442	2.35	0.012268
Chromosome 6 open reading frame 155	BE500942	2.35	0.02984
CDC20 cell division cycle 20 homolog (S. cerevisiae)	NM_001255	2.36	0.002795
PHD finger protein 19	BE544837	2.36	0.004485
ARG99 protein	BF109231	2.37	0.002605
gb:AW139393 /DB_XREF=gi:6144111 /DB_XREF=UI-H-BI1-adq-c-06-0-UI.s1 /CLONE=IMAGE:2717578 /FEA=EST /CNT=14 /TID=Hs.62954.2 /TIER=Stack /STK=11 /UG=Hs.62954 /LL=2495 /UG_GENE=FTH1 /UG_TITLE=ferritin, heavy polypeptide 1	AW139393	2.37	0.002988
retinoic acid receptor responder (tazarotene induced) 2	BC000069	2.39	0.005649
suppressor of cytokine signaling 1	AB005043	2.4	0.063822
hypothetical LOC388610	AA037766	2.4	0.001844

neurexin 3	NM_004796	2.41	0.010341
exocyst complex component 7	AK023832	2.47	0.011497
hypothetical protein MGC45871	BG036514	2.47	0.001233
glutaredoxin (thioltransferase)	NM_002064	2.48	0.002461
stathmin-like 3	AL353715	2.51	0.000756
regeneration associated muscle protease	AI671186	2.52	0.000086
T-box 3 (ulnar mammary syndrome)	AI806338	2.53	0.003524
ribonucleotide reductase M2 polypeptide	BE966236	2.54	0.002883
glutaredoxin (thioltransferase)	AF162769	2.54	0.008125
BH-protocadherin (brain-heart)	NM_002589	2.55	0.041771
Homo sapiens, clone IMAGE:4865533, mRNA	AA622837	2.55	0.002441
deiodinase, iodothyronine, type II	NM_013989	2.58	0.012863
tumor necrosis factor (ligand) superfamily, member 4 (tax-transcriptionally activated glycoprotein 1, 34kDa)	NM_003326	2.58	0.001359
heat shock 70kDa protein 2	U56725	2.6	0.00041
hypothetical protein MGC16121	BE048571	2.6	0.017238
transcription factor AP-2 alpha (activating enhancer binding protein 2 alpha)	BF343007	2.61	0.000313
renin	NM_000537	2.63	0.045025
chromosome 1 open reading frame 24	AF288391	2.64	0.025609
Hypothetical protein MGC16121	AA234096	2.65	0.01469
odd-skipped related 2 (Drosophila)	AI811298	2.67	0.006123
gb:AV699565 /DB_XREF=gi:10301536 /DB_XREF=AV699565 /CLONE=GKCDPD06 /FEA=EST /CNT=9 /TID=Hs.128065.0 /TIER=Stack /STK=9 /UG=Hs.128065 /UG_TITLE=ESTs	AV699565	2.67	0.003202
caspase 1, apoptosis-related cysteine protease (interleukin 1, beta, convertase) /// CARD only protein	NM_052889	2.67	0.012445
interferon-induced protein with tetratricopeptide repeats 1 /// interferon-induced protein with tetratricopeptide repeats 1	NM_001548	2.68	0.014462
CDNA clone IMAGE:6025865, partial cds	AA156240	2.72	0.011709
KIAA1576 protein	AA156723	2.72	0.000919
homeo box A11	H94842	2.73	0.000994
KIAA1912 protein	AI376433	2.73	0.005538
serine (or cysteine) proteinase inhibitor, clade B (ovalbumin), member 7	NM_003784	2.74	0.003429
four jointed box 1 (Drosophila)	NM_014344	2.74	0.000207
opioid growth factor receptor-like 1	NM_024576	2.75	0.003979
hairy and enhancer of split 1, (Drosophila)	NM_005524	2.76	0.000009

endothelial differentiation, sphingolipid G-protein-coupled receptor, 1	NM_001400	2.78	0.003053
caspase recruitment domain family, member 9	NM_022352	2.79	0.000837
Pre-B-cell leukemia transcription factor 1	AL049381	2.81	0.002159
microphthalmia-associated transcription factor	NM_000248	2.83	0.000974
interferon-induced protein with tetratricopeptide repeats 2	AA131041	2.83	0.031536
hypothetical protein FLJ31166	BE301029	2.86	0.006563
ST6 (alpha-N-acetyl-neuraminyl-2,3-beta-galactosyl-1,3)-N-acetylgalactosaminide alpha-2,6-sialyltransferase 5	AF131837	2.88	0.000976
phorbol-12-myristate-13-acetate-induced protein 1	NM_021127	2.9	0.000931
endothelial differentiation, sphingolipid G-protein-coupled receptor, 3	AA534817	2.9	0.01875
gb:AA167449 /DB_XREF=gi:1745836 /DB_XREF=zp08h11.s1 /CLONE=IMAGE:595845 /FEA=mRNA /CNT=416 /TID=Hs.83623.2 /TIER=Stack /STK=59 /UG=Hs.83623 /LL=9970 /UG_GENE=NR1I3 /UG_TITLE=nuclear receptor subfamily 1, group I, member 3	AA167449	2.93	0.000936
hypothetical protein FLJ20245	NM_017723	2.97	0.001263
T-box 3 (ulnar mammary syndrome)	NM_016569	2.97	0.001002
spondin 2, extracellular matrix protein	NM_012445	3.08	0.011901
Leukocyte-derived arginine aminopeptidase	BE889628	3.19	0.002461
CARD only protein	NM_052889	3.27	0.001887
gremlin 1 homolog, cysteine knot superfamily (Xenopus laevis)	NM_013372	3.3	0.001674
gb:AV646597 /DB_XREF=gi:9867611 /DB_XREF=AV646597 /CLONE=GLCAPB04 /FEA=EST /CNT=57 /TID=Hs.51615.0 /TIER=Stack /STK=34 /UG=Hs.51615 /UG_TITLE=ESTs, Weakly similar to ALU7_HUMAN ALU SUBFAMILY SQ SEQUENCE CONTAMINATION WARNING ENTRY (H.sapiens)	AV646597	3.34	0.000149
gremlin 1 homolog, cysteine knot superfamily (Xenopus laevis)	AF154054	3.36	0.000019
interferon-induced protein with tetratricopeptide repeats 3	AI075407	3.38	0.002065
nidogen (enactin)	BF940043	3.39	0.000113
keratin 19	NM_002276	3.4	0.001156
nuclear receptor subfamily 2, group F, member 2	AV703465	3.42	0.001717
nuclear receptor subfamily 2, group F, member 2	M64497	3.42	0.000041
aldehyde dehydrogenase 1 family, member A1	NM_000689	3.42	0.009298
RNA binding motif protein 24	AI677701	3.45	0.000673
coagulation factor II (thrombin) receptor-like 2	AI378647	3.5	0.008675
microphthalmia-associated transcription factor	AL117653	3.52	0.0035
gb:AV699347 /DB_XREF=gi:10301318 /DB_XREF=AV699347 /CLONE=GKCBPH07 /FEA=EST /CNT=18 /TID=Hs.83623.3 /TIER=Stack /STK=13 /UG=Hs.83623 /LL=9970 /UG_GENE=NR1I3 /UG_TITLE=nuclear receptor subfamily 1, group I, member 3	AV699347	3.57	0.000353

oxytocin receptor	NM_000916	3.62	0.000657
nuclear receptor subfamily 2, group F, member 2	AL037401	3.62	0.00031
neurofilament 3 (150kDa medium)	NM_005382	3.65	0.000173
ST6 (alpha-N-acetyl-neuraminyl-2,3-beta-galactosyl-1,3)-N-acetylgalactosaminide alpha-2,6-sialyltransferase 5 /// ST6 (alpha-N-acetyl-neuraminyl-2,3-beta-galactosyl-1,3)-N-acetylgalactosaminide alpha-2,6-sialyltransferase 5	NM_030965	3.7	0.000038
gb:AA628440 /DB_XREF=gi:2540827 /DB_XREF=af26f02.s1 /CLONE=IMAGE:1032795 /FEA=mRNA /CNT=416 /TID=Hs.83623.2 /TIER=Stack /STK=70 /UG=Hs.83623 /LL=9970 /UG_GENE=NR1I3 /UG_TITLE=nuclear receptor subfamily 1, group I, member 3	AA628440	3.7	0.000227
G protein-coupled receptor 126	AL033377	3.71	0.001295
mannan-binding lectin serine protease 1 (C4/C2 activating component of Ra-reactive factor)	AI274095	3.75	0.00758
cathepsin C	NM_001814	3.81	0.000301
hypothetical protein FLJ11127	NM_019018	3.81	0.000751
5-hydroxytryptamine (serotonin) receptor 2B	NM_000867	3.82	0.000725
homeo box D10	AW299531	4.13	0.00142
integrin, alpha 6	NM_000210	4.18	0.000506
forkhead box F1	NM_001451	4.3	0.002724
neurofilament, light polypeptide 68kDa	AL537457	4.37	0.000028
C-type lectin domain family 2, member B	BC005254	4.5	0.006642
coagulation factor III (thromboplastin, tissue factor)	NM_001993	4.58	0.003834
response gene to complement 32	NM_014059	4.59	0.014918
gb:AW263497 /DB_XREF=gi:6640313 /DB_XREF=xn80a06.x1 /CLONE=IMAGE:2700754 /FEA=EST /CNT=6 /TID=Hs.97774.0 /TIER=ConsEnd /STK=3 /UG=Hs.97774 /UG_TITLE=ESTs	AW263497	4.61	0.003856
forkhead box L2	NM_023067	4.87	0.000092
cathepsin C	AI246687	4.99	0.00003
GATA binding protein 2	AL563460	5.05	0.000409
keratin 18	NM_000224	5.25	0.000168
cathepsin C	AI246687	5.32	0.000084
matrix metalloproteinase 1 (interstitial collagenase)	NM_002421	5.81	0.000064
matrix metalloproteinase 3 (stromelysin 1, progelatinase)	NM_002422	6.17	0.000085
somatostatin receptor 1	R62424	6.19	0.001146

Supplementary Table 2: Lists of genes differentially expressed between bone Marrow MSCs and placenta MSCs.

Genes overexpressed in BMMSCs as compared to PlMSCs			
**Gene name**	**Accession Nb**	**Fold increase**	**P value**
cytochrome P450, family 1, subfamily B, polypeptide 1	NM_000104	7.73	0.003322
cytochrome P450, family 1, subfamily B, polypeptide 1	AU154504	6.75	0.001555
elastin (supravalvular aortic stenosis, Williams-Beuren syndrome)	AA479278	6.04	0.000888
cytochrome P450, family 1, subfamily B, polypeptide 1	AU144855	5.95	0.000071
proenkephalin	NM_006211	5.87	0.000088
EGF-containing fibulin-like extracellular matrix protein 1	AI826799	5.76	0.001122
potassium channel tetramerisation domain containing 12	AI718937	5.58	0.012338
prostaglandin I2 (prostacyclin) synthase /// prostaglandin I2 (prostacyclin) synthase	NM_000961	5.56	0.01359
hyaluronan and proteoglycan link protein 1	U43328	5.35	0.000069
tissue inhibitor of metalloproteinase 3 (Sorsby fundus dystrophy, pseudoinflammatory)	NM_000362	5.26	0.000915
iroquois homeobox protein 3	AI681917	5.23	0.000791
pentraxin-related gene, rapidly induced by IL-1 beta	NM_002852	5.22	0.009695
tissue inhibitor of metalloproteinase 3 (Sorsby fundus dystrophy, pseudoinflammatory)	BF347089	4.89	0.000555
filaggrin	AL356504	4.85	0.002765
ribosomal protein S4, Y-linked 1	NM_001008	4.78	0.004168
UDP-N-acetyl-alpha-D-galactosamine:polypeptide N-acetylgalactosaminyltransferase 5 (GalNAc-T5)	AK022198	4.78	0.006856
ankyrin 3, node of Ranvier (ankyrin G)	NM_020987	4.75	0.006564
tissue inhibitor of metalloproteinase 3 (Sorsby fundus dystrophy, pseudoinflammatory)	U67195	4.74	0.000003
phosphatidic acid phosphatase type 2B	AV725664	4.74	0.006599
paired-like homeodomain transcription factor 2	NM_000325	4.7	0.001638
Hyaluronan and proteoglycan link protein 1	AU144114	4.7	0.003921
EGF-containing fibulin-like extracellular matrix protein 1	NM_004105	4.49	0.000533
fibronectin type III domain containing 1	AI345957	4.4	0.000158
potassium channel tetramerisation domain containing 12 /// potassium channel tetramerisation domain containing 12	AA551075	4.39	0.003519
CDNA: FLJ23131 fis, clone LNG08502	AK026784	4.19	0.00245
receptor tyrosine kinase-like orphan receptor 1	NM_005012	4.18	0.010172

prostaglandin E synthase	NM_004878	4.1	0.000385
paired related homeobox 2	NM_016307	4.1	0.000147
keratin associated protein 1-1 /// keratin associated protein 1-1	NM_030967	4.1	0.00091
tissue inhibitor of metalloproteinase 3 (Sorsby fundus dystrophy, pseudoinflammatory)	AW338933	4.09	0.001924
integrin, beta-like 1 (with EGF-like repeat domains)	NM_004791	4.09	0.001551
gb:AL359052.1 /DB_XREF=gi:8518175 /FEA=mRNA /CNT=13 /TID=Hs.311054.0 /TIER=ConsEnd /STK=5 /UG=Hs.311054 /DEF=Homo sapiens mRNA full length insert cDNA clone EUROIMAGE 1968422. /PROD=ITGBL1, integrin beta-like 1	AL359052	4.04	0.003044
Integrin, beta-like 1 (with EGF-like repeat domains)	AI753143	4.03	0.000777
crystallin, alpha B	AF007162	4.01	0.001219
importin 9	AI659533	3.94	0.001105
transforming growth factor, beta-induced, 68kDa	NM_000358	3.93	0.010403
aldehyde dehydrogenase 1 family, member A3	NM_000693	3.9	0.004309
GATA binding protein 6	D87811	3.84	0.003577
microfibrillar associated protein 5	AW665892	3.82	0.003055
dapper homolog 1, antagonist of beta-catenin (xenopus)	NM_016651	3.82	0.007952
CDNA FLJ31059 fis, clone HSYRA2000832	AA418028	3.8	0.000001
phosphatidic acid phosphatase type 2B	AB000889	3.79	0.005991
major histocompatibility complex, class II, DP alpha 1	M27487	3.78	0.002291
hyaluronan and proteoglycan link protein 1	NM_001884	3.75	0.000019
hypothetical protein DKFZp434L142	AF260333	3.74	0.001039
ABI gene family, member 3 (NESH) binding protein	AB056106	3.72	0.000094
UDP-N-acetyl-alpha-D-galactosamine:polypeptide N-acetylgalactosaminyltransferase 1 (GalNAc-T1)	BC038440	3.72	0.000437
decorin	AF138302	3.63	0.004041
integrin, alpha 11	AF109681	3.62	0.005288
endothelial cell-specific molecule 1	NM_007036	3.54	0.13248
serine (or cysteine) proteinase inhibitor, clade E (nexin, plasminogen activator inhibitor type 1), member 2	AL541302	3.52	0.024069
cyclin-dependent kinase inhibitor 2B (p15, inhibits CDK4)	AW444761	3.5	0.001621
supervillin	NM_003174	3.49	0.001763
gb:BF002195 /DB_XREF=gi:10702470 /DB_XREF=7h23h07.x1 /CLONE=IMAGE:3316861 /FEA=EST /CNT=8 /TID=Hs.131624.0 /TIER=ConsEnd /STK=6 /UG=Hs.131624 /UG_TITLE=ESTs	BF002195	3.49	0.000004
UDP-N-acetyl-alpha-D-galactosamine:polypeptide N-acetylgalactosaminyltransferase 1 (GalNAc-T1)	AV692127	3.47	0.001518
DEAD (Asp-Glu-Ala-Asp) box polypeptide 3, Y-linked	NM_004660	3.47	0.002768
aldo-keto reductase family 1, member C3 (3-alpha hydroxysteroid dehydrogenase, type II)	AB018580	3.45	0.026754

phosphatidic acid phosphatase type 2B	AA628586	3.43	0.002066
microfibrillar associated protein 5	AW665892	3.43	0.002064
prostaglandin-endoperoxide synthase 2 (prostaglandin G/H synthase and cyclooxygenase)	NM_000963	3.42	0.025127
hypothetical protein LOC283824	AI693140	3.42	0.049877
decorin	AF138300	3.41	0.001438
decorin	AF138303	3.39	0.001012
hypothetical protein FLJ14054	NM_024563	3.39	0.036858
UDP-N-acetyl-alpha-D-galactosamine:polypeptide N-acetylgalactosaminyltransferase 1 (GalNAc-T1)	U41514	3.37	0.000708
ectonucleotide pyrophosphatase/phosphodiesterase 2 (autotaxin)	L35594	3.35	0.000112
CDNA clone IMAGE:4152983, partial cds	BF344237	3.31	0.000021
vascular cell adhesion molecule 1	NM_001078	3.29	0.000728
tumor protein D52-like 1	NM_003287	3.25	0.016412
KIAA0746 protein	AB018289	3.25	0.005595
dehydrogenase/reductase (SDR family) member 3	NM_004753	3.21	0.00583
insulin-like growth factor binding protein 2, 36kDa	NM_000597	3.21	0.000488
Homo sapiens, clone IMAGE:4794726, mRNA	BF513121	3.2	0.005868
gb:AW964972 /DB_XREF=gi:8154808 /DB_XREF=EST377045 /FEA=EST /CNT=66 /TID=Hs.48516.1 /TIER=Stack /STK=50 /UG=Hs.48516 /UG_TITLE=ESTs	AW964972	3.2	0.000353
hemicentin	BF446673	3.2	0.001169
pregnancy-associated plasma protein A, pappalysin 1	BF107618	3.18	0.001288
UDP-N-acetyl-alpha-D-galactosamine:polypeptide N-acetylgalactosaminyltransferase 1 (GalNAc-T1)	NM_020474	3.17	0.002355
chondroitin sulfate proteoglycan 2 (versican)	BF218922	3.17	0.017853
SMAD, mothers against DPP homolog 6 (Drosophila)	NM_005585	3.16	0.003863
cartilage oligomeric matrix protein	NM_000095	3.15	0.001844
lysyl oxidase	L16895	3.15	0.022313
pregnancy-associated plasma protein A, pappalysin 1	BF107618	3.15	0.002958
serine/threonine kinase 38 like	AW779556	3.14	0.014126
EGF, latrophilin and seven transmembrane domain containing 1	NM_022159	3.14	0.013261
ankyrin repeat domain 1 (cardiac muscle)	NM_014391	3.13	0.032685
Similar to circumsporozoite protein precursor - Plasmodium brasilianum	H10408	3.13	0.00286
adducin 3 (gamma)	NM_019903	3.11	0.005088
decorin	AI281593	3.11	0.004863
microfibrillar associated protein 5	U37283	3.11	0.000945
chondroitin sulfate proteoglycan 2 (versican)	NM_004385	3.1	0.01905

solute carrier family 14 (urea transporter), member 1 (Kidd blood group)	NM_015865	3.1	0.00001
adducin 3 (gamma)	AI763123	3.09	0.000066
adducin 3 (gamma)	AI818488	3.07	0.003946
pregnancy-associated plasma protein A, pappalysin 1	AI110886	3.07	0.004067
ankylosis, progressive homolog (mouse)	AA854943	3.04	0.000857
placental growth factor, vascular endothelial growth factor-related protein	BC001422	3.02	0.003553
adducin 3 (gamma)	BE545756	3.01	0.000401
Solute carrier family 14 (urea transporter), member 1 (Kidd blood group)	BE673587	3.01	0.000977
ATP-binding cassette, sub-family A (ABC1), member 8	NM_007168	2.99	0.000622
early B-cell factor 3	AL354950	2.99	0.00078
prostaglandin E synthase	AF010316	2.96	0.000405
lumican	NM_002345	2.94	0.007545
chemokine (C-X-C motif) ligand 12 (stromal cell-derived factor 1)	U19495	2.94	0.03488
chitinase 3-like 1 (cartilage glycoprotein-39)	M80927	2.92	0.083965
KIAA1199	AB033025	2.92	0.021069
Forkhead box C2 (MFH-1, mesenchyme forkhead 1)	BF446723	2.92	0.001247
CD74 antigen (invariant polypeptide of major histocompatibility complex, class II antigen-associated)	K01144	2.91	0.001428
KIAA0367	AB002365	2.91	0.000141
v-maf musculoaponeurotic fibrosarcoma oncogene homolog (avian)	AF055376	2.9	0.000137
insulin-like growth factor binding protein 3	BF340228	2.9	0.000304
jagged 1 (Alagille syndrome)	U73936	2.88	0.002697
chromosome 7 open reading frame 10	NM_024728	2.87	0.000623
Full-length cDNA clone CS0DD009YB17 of Neuroblastoma Cot 50-normalized of Homo sapiens (human)	AK000776	2.87	0.000056
SMAD, mothers against DPP homolog 6 (Drosophila)	AI193899	2.86	0.002294
Fibronectin 1	AI333596	2.86	0.001827
procollagen C-endopeptidase enhancer 2	NM_013363	2.85	0.00025
jagged 1 (Alagille syndrome)	U77914	2.84	0.000002
UDP-N-acetyl-alpha-D-galactosamine:polypeptide N-acetylgalactosaminyltransferase 5 (GalNAc-T5)	AI633503	2.84	0.000042
similar to expressed sequence AI593442	H24398	2.84	0.00141
interleukin 6 (interferon, beta 2)	NM_000600	2.83	0.000275
pregnancy-associated plasma protein A, pappalysin 1	BG434272	2.81	0.000003
chondroitin sulfate proteoglycan 2 (versican) /// chondroitin sulfate proteoglycan 2 (versican)	R94644	2.79	0.002415
gb:AW044286 /DB_XREF=gi:5904815 /DB_XREF=wv67c06.x1 /CLONE=IMAGE:2534602 /FEA=EST /CNT=19 /TID=Hs.174134.2 /TIER=Stack /STK=18 /UG=Hs.174134 /LL=51204 /UG_GENE=LOC51204	AW044286	2.79	0.000927

/UG_TITLE=clone HQ0477 PRO0477p			
latrophilin 2	NM_012302	2.78	0.003297
chitinase 3-like 1 (cartilage glycoprotein-39)	M80927	2.78	0.08551
putative small membrane protein NID67	AF313413	2.78	0.016162
Collagen, type VIII, alpha 1	AL359062	2.78	0.001695
DEP domain containing 6	NM_022783	2.77	0.000692
Hyaluronan and proteoglycan link protein 1	AI760252	2.75	0.000486
kelch-like 13 (Drosophila)	AB037730	2.73	0.004358
cadherin 6, type 2, K-cadherin (fetal kidney)	AU151483	2.72	0.000662
pregnancy-associated plasma protein A, pappalysin 1	AA148534	2.71	0.000262
stanniocalcin 2	AI435828	2.71	0.00133
CD24 antigen (small cell lung carcinoma cluster 4 antigen)	AK000168	2.71	0.016751
Ubiquitin specific protease 9, Y-linked (fat facets-like, Drosophila)	AV681765	2.7	0.010913
zinc finger protein 423	AW149417	2.68	0.007633
Kruppel-like factor 4 (gut)	BF514079	2.68	0.001744
transforming growth factor, beta receptor I (activin A receptor type II-like kinase, 53kDa)	AA604375	2.68	0.01058
hypothetical gene supported by BC009385	AL520272	2.68	0.000025
chromosome 5 open reading frame 13	NM_004772	2.66	0.016683
CDNA FLJ44429 fis, clone UTERU2015653	AI088063	2.66	0.00277
Homo sapiens, clone IMAGE:4837650, mRNA, partial cds	AI862542	2.66	0.000036
hypothetical LOC389048	BG285837	2.66	0.008768
insulin-like growth factor binding protein 3	M31159	2.65	0.006836
collagen triple helix repeat containing 1	AA584310	2.65	0.000233
LIM domain only 2 (rhombotin-like 1)	NM_005574	2.64	0.002421
heat shock 27kDa protein family, member 7 (cardiovascular)	NM_014424	2.64	0.003171
core 1 synthase, glycoprotein-N-acetylgalactosamine 3-beta-galactosyltransferase, 1	NM_020156	2.64	0.006474
glutaminase	NM_014905	2.61	0.000804
fibronectin 1	X02761	2.61	0.021412
fibronectin 1	AF130095	2.6	0.021926
chondroitin sulfate proteoglycan 2 (versican)	BF590263	2.59	0.025301
Full-length cDNA clone CS0DI014YH21 of Placenta Cot 25-normalized of Homo sapiens (human)	BF058422	2.59	0.030234
cytoglobin	AL513673	2.59	0.000052
biglycan /// serologically defined colon cancer antigen 33	AA845258	2.58	0.027342
CDNA FLJ26539 fis, clone KDN09310	AW025579	2.57	0.027809

netrin 4	AF278532	2.57	0.005984
fibroblast growth factor 7 (keratinocyte growth factor)	NM_002009	2.56	0.032277
fibronectin 1 /// fibronectin 1	BC005858	2.56	0.022104
fibronectin 1	AK026737	2.56	0.021662
ankyrin repeat domain 29	AI307802	2.56	0.000808
periostin, osteoblast specific factor	AY140646	2.56	0.005378
heme oxygenase (decycling) 1	NM_002133	2.55	0.000054
CD24 antigen (small cell lung carcinoma cluster 4 antigen)	L33930	2.55	0.009463
BMP and activin membrane-bound inhibitor	NM_012342	2.54	0.0016
ependymin related protein 1 (zebrafish)	BC000686	2.53	0.000081
gb:AA954994 /DB_XREF=gi:3118689 /DB_XREF=op24f03.s1 /CLONE=IMAGE:1577789 /FEA=EST /CNT=15 /TID=Hs.237396.1 /TIER=ConsEnd /STK=1 /UG=Hs.237396 /UG_TITLE=ESTs	AA954994	2.52	0.000399
EGF-like repeats and discoidin I-like domains 3	AA053711	2.51	0.030477
Glypican 6	AI651255	2.5	0.034156
glutaminase	AF097493	2.49	0.000005
CD24 antigen (small cell lung carcinoma cluster 4 antigen)	AA761181	2.48	0.004905
complement factor H /// complement factor H-related 1	X56210	2.48	0.005899
biglycan	BC002416	2.47	0.035548
lysyl oxidase	NM_002317	2.46	0.006742
Full length insert cDNA clone ZD77F06	AI655611	2.45	0.000456
zinc finger protein 503	AA603467	2.45	0.00164
hypothetical protein FLJ38725	AI659800	2.45	0.000979
periostin, osteoblast specific factor	AY140646	2.45	0.004691
Ifapsoriasin	BC029685	2.45	0.004666
phosphoglucomutase 1	NM_002633	2.44	0.000839
protein phosphatase 1, regulatory (inhibitor) subunit 3C	N26005	2.43	0.000283
phospholipase A2, group IVA (cytosolic, calcium-dependent)	M68874	2.43	0.005079
NACHT, leucine rich repeat and PYD (pyrin domain) containing 1	NM_021730	2.43	0.009505
major histocompatibility complex, class II, DR beta 1	AJ297586	2.42	0.000434
ankyrin 2, neuronal	BF726212	2.41	0.013105
EPH receptor A3	AF213459	2.4	0.003771
interferon-induced protein 44	NM_006417	2.4	0.000148
fibronectin 1	AJ276395	2.4	0.000259
LOC440156	BE738279	2.39	0.000071

secretory protein LOC284013	AA977975	2.38	0.000602
Jagged 1 (Alagille syndrome)	AI457817	2.36	0.002169
gb:AB020690.1 /DB_XREF=gi:4240254 /GEN=KIAA0883 /FEA=FLmRNA /CNT=85 /TID=Hs.7782.0 /TIER=FL+Stack /STK=20 /UG=Hs.7782 /LL=10687 /DEF=Homo sapiens mRNA for KIAA0883 protein, complete cds. /PROD=KIAA0883 protein /FL=gb:NM_007257.1 gb:AB020690.1 gb:AF286487.1	AB020690	2.35	0.002511
aggrecan 1 (chondroitin sulfate proteoglycan 1, large aggregating proteoglycan, antigen identified by monoclonal antibody A0122)	NM_013227	2.34	0.000363
Ras association (RalGDS/AF-6) and pleckstrin homology domains 1	AA194149	2.34	0.03458
hypothetical protein FLJ14834	AW084730	2.34	0.015983
gap junction protein, alpha 1, 43kDa (connexin 43)	NM_000165	2.33	0.001489
hyaluronan synthase 1	NM_001523	2.33	0.00013
lysyl oxidase-like 3	AI917716	2.33	0.000034
Hyaluronan synthase 2	AI374739	2.33	0.133892
aldo-keto reductase family 1, member C2 (dihydrodiol dehydrogenase 2; bile acid binding protein; 3-alpha hydroxysteroid dehydrogenase, type III) /// aldo-keto reductase family 1, member C2 (dihydrodiol dehydrogenase 2; bile acid binding protein; 3-alpha hydroxysteroid dehydrogenase, type III)	M33376	2.32	0.012668
natriuretic peptide receptor C/guanylate cyclase C (atrionatriuretic peptide receptor C)	AI628360	2.32	0.006188
tumor necrosis factor, alpha-induced protein 3	NM_006290	2.31	0.034927
hephaestin	NM_014799	2.31	0.000294
growth arrest-specific 1	NM_002048	2.3	0.000025
KIAA1671 protein	AB051458	2.3	0.00827
Transcribed locus	BG236273	2.3	0.000147
nicotinamide N-methyltransferase	NM_006169	2.29	0.005685
connective tissue growth factor	M92934	2.29	0.056539
hepatocyte growth factor (hepapoietin A; scatter factor)	X16323	2.29	0.039711
vascular endothelial growth factor	AF022375	2.29	0.002075
LOC440156	AL530703	2.29	0.008098
midline 1 (Opitz/BBB syndrome)	NM_000381	2.28	0.009283
adiponutrin	AK025665	2.27	0.000306
chromosome 10 open reading frame 116	NM_006829	2.26	0.036518
cell cycle progression 1	NM_004748	2.26	0.015605
nicotinamide N-methyltransferase	NM_006169	2.23	0.0096
special AT-rich sequence binding protein 1 (binds to nuclear matrix/scaffold-associating DNA's)	NM_002971	2.23	0.000248
phosphodiesterase 4D interacting protein (myomegalin)	AB042557	2.23	0.012388
periostin, osteoblast specific factor	D13665	2.22	0.005131

glutathione peroxidase 7	AA406605	2.22	0.001401
fibronectin 1	AJ276395	2.22	0.000166
Plexin A4	AL117427	2.22	0.001217
insulin-like growth factor binding protein 4	NM_001552	2.21	0.023921
chondroitin sulfate proteoglycan 2 (versican)	D32039	2.21	0.00362
frizzled homolog 8 (Drosophila)	AW340311	2.21	0.084024
MRNA; cDNA DKFZp313C0240 (from clone DKFZp313C0240)	H16409	2.21	0.00106
EPH receptor A5	BE218107	2.21	0.00208
Core 1 synthase, glycoprotein-N-acetylgalactosamine 3-beta-galactosyltransferase, 1	AI672489	2.18	0.024178
scinderin	NM_033128	2.18	0.000882
4-aminobutyrate aminotransferase	AF237813	2.16	0.006358
enabled homolog (Drosophila)	NM_018212	2.14	0.007709
Doublecortin and CaM kinase-like 1	AI129626	2.13	0.005105
reversion-inducing-cysteine-rich protein with kazal motifs	NM_021111	2.12	0.013573
gb:BF221547 /DB_XREF=gi:11128724 /DB_XREF=7o69f05.x1 /CLONE=IMAGE:3579416 /FEA=EST /CNT=54 /TID=Hs.16578.0 /TIER=Stack /STK=31 /UG=Hs.16578 /UG_TITLE=ESTs	BF221547	2.12	0.02871
Xg blood group (pseudoautosomal boundary-divided on the X chromosome)	AF380356	2.12	0.005557
stanniocalcin 2	BC000658	2.11	0.000104
cadherin 11, type 2, OB-cadherin (osteoblast)	D21254	2.11	0.008236
Similar to CG9996-PA	AI004009	2.11	0.002424
microtubule-associated protein 1B	AA554833	2.11	0.004541
cytokine receptor-like factor 1	NM_004750	2.1	0.00021
transmembrane, prostate androgen induced RNA	AL035541	2.1	0.034116
KIAA1644 protein	BF516072	2.09	0.000149
serine (or cysteine) proteinase inhibitor, clade F (alpha-2 antiplasmin, pigment epithelium derived factor), member 1	NM_002615	2.08	0.013844
meningioma (disrupted in balanced translocation) 1	NM_002430	2.08	0.001493
gb:NM_021647.1 /DB_XREF=gi:11067364 /GEN=KIAA0626 /FEA=FLmRNA /CNT=50 /TID=Hs.178121.0 /TIER=FL+Stack /STK=20 /UG=Hs.178121 /LL=9848 /DEF=Homo sapiens KIAA0626 gene product (KIAA0626), mRNA. /PROD=KIAA0626 gene product /FL=gb:AB014526.1 gb:NM_021647.1	NM_021647	2.08	0.005642
Transcribed locus	BF513800	2.08	0.004004
KIAA1102 protein	AK026815	2.07	0.004642
tumor differentially expressed 2-like	AA872583	2.07	0.01738
Ras association (RalGDS/AF-6) and pleckstrin homology domains 1	AA194149	2.07	0.017083

glycerol-3-phosphate acyltransferase, mitochondrial	AB046780	2.07	0.000705
pleckstrin and Sec7 domain containing 3	NM_015310	2.06	0.003508
WAP four-disulfide core domain 1	NM_021197	2.06	0.003962
Homo sapiens, Similar to HERV-H LTR-associating 3, clone IMAGE:4830680, mRNA	BC031956	2.06	0.014378
leukemia inhibitory factor (cholinergic differentiation factor)	NM_002309	2.05	0.00309
Transcribed locus	AI761573	2.05	0.023009
keratin associated protein 1-5	AJ406928	2.05	0.004435
selenoprotein P, plasma, 1	NM_005410	2.04	0.004461
interleukin 1 receptor, type I	NM_000877	2.04	0.009638
eukaryotic translation initiation factor 1A, Y-linked	BC005248	2.04	0.006878
clusterin (complement lysis inhibitor, SP-40,40, sulfated glycoprotein 2, testosterone-repressed prostate message 2, apolipoprotein J)	AI982754	2.03	0.029207
Serine (or cysteine) proteinase inhibitor, clade E (nexin, plasminogen activator inhibitor type 1), member 2	AA703280	2.03	0.008867
family with sequence similarity 43, member A	AW264102	2.02	0.032255
Similar to Ras suppressor protein 1 (Rsu-1) (RSP-1)	AW167298	2.02	0.007415
Hypothetical protein LOC201895	AI824013	2.01	0.016648
phosphodiesterase 7B	AI638433	2.01	0.00081
aldehyde dehydrogenase 1 family, member L2	AI654224	2.01	0.091881
Collagen, type XII, alpha 1	AU146651	2.01	0.018747
similar to AVLV472	BC021680	2.01	0.001958
midline 1 (Opitz/BBB syndrome)	BE967532	2	0.015403
aldo-keto reductase family 1, member C1 (dihydrodiol dehydrogenase 1; 20-alpha (3-alpha)-hydroxysteroid dehydrogenase)	NM_001353	2	0.033326
bone morphogenetic protein 1	NM_001199	2	0.001788
docking protein 1, 62kDa (downstream of tyrosine kinase 1)	AF035299	2	0.001049
Similar to CG9996-PA	AW025980	2	0.000307

Genes overexpressed in Pl-MSCs as compared to BM-MSCs			
**Gene name**	**Accession Nb**	**Fold increase**	**P value**
membrane metallo-endopeptidase (neutral endopeptidase, enkephalinase, CALLA, CD10)	AI433463	2	0.043038
thyrotropin-releasing hormone degrading ectoenzyme	NM_013381	2	0.012203
S100 calcium binding protein A4 (calcium protein, calvasculin, metastasin, murine placental homolog)	NM_002961	2.01	0.040158
ZW10 interactor	NM_007057	2.01	0.000487
family with sequence similarity 54, member A	AL138828	2.02	0.00618
fatty acid binding protein 4, adipocyte	NM_001442	2.04	0.008839
monoglyceride lipase	BG168471	2.04	0.026765
chromosome 20 open reading frame 129	BC001068	2.04	0.000062
Transcribed locus	BF476403	2.06	0.00691
MCM7 minichromosome maintenance deficient 7 (S. cerevisiae)	D55716	2.08	0.000384
hypothetical protein FLJ10156	BC005004	2.08	0.007833
kinesin family member 2C	U63743	2.09	0.000788
putative homeodomain transcription factor 2	AL136883	2.09	0.001359
chromosome 8 open reading frame 4	NM_020130	2.09	0.057571
spindle pole body component 25 homolog (S. cerevisiae)	AF225416	2.1	0.000726
geminin, DNA replication inhibitor	NM_015895	2.1	0.000739
asp (abnormal spindle)-like, microcephaly associated (Drosophila)	NM_018123	2.1	0.019218
tetraspanin 5 /// tetraspanin 5	AF065389	2.11	0.000351
exocyst complex component 7	AK023832	2.12	0.021592
cell division cycle associated 7 /// cell division cycle associated 7	AY029179	2.13	0.010939
Integrin, alpha 2 (CD49B, alpha 2 subunit of VLA-2 receptor)	N95414	2.13	0.050357
deiodinase, iodothyronine, type II	NM_013989	2.14	0.009561
PDZ and LIM domain 1 (elfin)	BC000915	2.14	0.000945
collagen, type XIII, alpha 1	M33653	2.14	0.009877
apolipoprotein B mRNA editing enzyme, catalytic polypeptide-like 3B	NM_004900	2.15	0.003126
MCM10 minichromosome maintenance deficient 10 (S. cerevisiae)	AL136840	2.15	0.010126
FERM domain containing 4A	AW515645	2.15	0.005929
aminoacylase 1-like 2	AI654133	2.15	0.018617
calponin 1, basic, smooth muscle	NM_001299	2.16	0.001544
monoglyceride lipase /// monoglyceride lipase	BC006230	2.16	0.036158

phorbol-12-myristate-13-acetate-induced protein 1	AI857639	2.19	0.0392
retinoic acid receptor responder (tazarotene induced) 2	BC000069	2.19	0.005615
tetraspanin 14 /// tetraspanin 14	NM_030927	2.19	0.000176
hypothetical protein MGC11324 /// hypothetical protein MGC11324	BC006236	2.19	0.013253
aldehyde oxidase 1	NM_001159	2.2	0.018708
insulin receptor substrate 2	BF700086	2.22	0.000439
hypothetical protein LOC146909	AA292789	2.22	0.000495
hypothetical LOC387763	AW276078	2.22	0.075617
suppressor of cytokine signaling 1	AB005043	2.23	0.065338
adaptor-related protein complex 1, sigma 2 subunit	AF251295	2.24	0.000603
hairy and enhancer of split 1, (Drosophila)	BE973687	2.24	0.003404
agrin	AK021586	2.24	0.039404
CD44 antigen (homing function and Indian blood group system)	AV700298	2.25	0.014558
sal-like 1 (Drosophila)	AU152837	2.25	0.04414
interleukin 11	NM_000641	2.27	0.225316
PHD finger protein 19	BE544837	2.27	0.005938
neurexin 3	NM_004796	2.28	0.002698
leucine rich repeat containing 17	NM_005824	2.29	0.000143
KIAA0186 gene product	NM_021067	2.29	0.002548
guanylate binding protein 3	AL136680	2.29	0.012075
hypothetical protein MGC45871	AI961778	2.29	0.000279
BH-protocadherin (brain-heart)	NM_002589	2.3	0.043911
E2F transcription factor 7	AI341146	2.3	0.021306
ribonucleotide reductase M2 polypeptide	BC001886	2.32	0.000006
regeneration associated muscle protease	AI671186	2.32	0.005964
homeo box A11	H94842	2.33	0.007392
transcription factor 19 (SC1)	BC002493	2.33	0.007071
churchill domain containing 1	BE568660	2.34	0.013767
CDNA clone IMAGE:6025865, partial cds	AA156240	2.35	0.006482
Homo sapiens, clone IMAGE:4865533, mRNA	AA622837	2.35	0.005141
CDC20 cell division cycle 20 homolog (S. cerevisiae)	NM_001255	2.36	0.001543
interferon-induced protein with tetratricopeptide repeats 1 /// interferon-induced protein with tetratricopeptide repeats 1	NM_001548	2.36	0.062663
glycoprotein hormones, alpha polypeptide	NM_000735	2.36	0.011827

Pre-B-cell leukemia transcription factor 1	AL049381	2.36	0.002676
collagen, type VII, alpha 1 (epidermolysis bullosa, dystrophic, dominant and recessive)	NM_000094	2.38	0.052994
solute carrier family 7 (cationic amino acid transporter, y+ system), member 2	AA876372	2.39	0.006668
elastin microfibril interfacer 2 /// elastin microfibril interfacer 2	AF270513	2.41	0.000444
tumor necrosis factor (ligand) superfamily, member 4 (tax-transcriptionally activated glycoprotein 1, 34kDa)	NM_003326	2.42	0.001132
agrin	AW008051	2.42	0.015497
Hypothetical protein MGC16121	AA234096	2.45	0.018547
ribonucleotide reductase M2 polypeptide	BE966236	2.46	0.00395
chromosome 1 open reading frame 24	AF288391	2.46	0.026045
interferon-induced protein with tetratricopeptide repeats 2	AA131041	2.47	0.023634
renin	NM_000537	2.49	0.095929
microphthalmia-associated transcription factor	NM_000248	2.49	0.013075
odd-skipped related 2 (Drosophila)	AI811298	2.49	0.00935
endothelial cell adhesion molecule	AL573851	2.49	0.003023
hypothetical protein MGC45871	BG036514	2.49	0.002966
Chromosome 6 open reading frame 155	BE500942	2.5	0.054195
hairy and enhancer of split 1, (Drosophila)	NM_005524	2.51	0.003673
glutathione S-transferase theta 1	NM_000853	2.51	0.015623
hypothetical protein MGC16121	BE048571	2.51	0.010197
transcription factor AP-2 alpha (activating enhancer binding protein 2 alpha)	BF343007	2.52	0.000221
Dedicator of cytokinesis 2	AI991459	2.53	0.032687
heat shock 70kDa protein 2	U56725	2.54	0.000369
aldehyde dehydrogenase 1 family, member A1	NM_000689	2.54	0.008716
uridine phosphorylase 1	NM_003364	2.57	0.014326
KIAA1912 protein	AI376433	2.57	0.001454
glutaredoxin (thioltransferase)	AF162769	2.59	0.006341
gb:AV699565 /DB_XREF=gi:10301536 /DB_XREF=AV699565 /CLONE=GKCDPD06 /FEA=EST /CNT=9 /TID=Hs.128065.0 /TIER=Stack /STK=9 /UG=Hs.128065 /UG_TITLE=ESTs	AV699565	2.59	0.01084
glutaredoxin (thioltransferase)	NM_002064	2.6	0.000033
gb:AW139393 /DB_XREF=gi:6144111 /DB_XREF=UI-H-BI1-adq-c-06-0-UI.s1 /CLONE=IMAGE:2717578 /FEA=EST /CNT=14 /TID=Hs.62954.2 /TIER=Stack /STK=11 /UG=Hs.62954 /LL=2495 /UG_GENE=FTH1 /UG_TITLE=ferritin, heavy polypeptide 1	AW139393	2.62	0.005458
endothelial differentiation, sphingolipid G-protein-coupled receptor, 3	AA534817	2.66	0.007023
L1 cell adhesion molecule	AI653981	2.69	0.007091

KIAA1576 protein	AA156723	2.69	0.000073
hypothetical protein FLJ31166	BE301029	2.69	0.002464
stathmin-like 3	AL353715	2.71	0.000026
opioid growth factor receptor-like 1	NM_024576	2.73	0.00142
T-box 3 (ulnar mammary syndrome)	AI806338	2.74	0.017697
hypothetical LOC388610	AA037766	2.76	0.001549
ST6 (alpha-N-acetyl-neuraminyl-2,3-beta-galactosyl-1,3)-N-acetylgalactosaminide alpha-2,6-sialyltransferase 5	AF131837	2.76	0.001248
caspase recruitment domain family, member 9	NM_022352	2.82	0.002695
four jointed box 1 (Drosophila)	NM_014344	2.83	0.001324
stanniocalcin 1	AI300520	2.84	0.068788
caspase 1, apoptosis-related cysteine protease (interleukin 1, beta, convertase) /// CARD only protein	NM_052889	2.85	0.008371
gb:AA167449 /DB_XREF=gi:1745836 /DB_XREF=zp08h11.s1 /CLONE=IMAGE:595845 /FEA=mRNA /CNT=416 /TID=Hs.83623.2 /TIER=Stack /STK=59 /UG=Hs.83623 /LL=9970 /UG_GENE=NR1I3 /UG_TITLE=nuclear receptor subfamily 1, group I, member 3	AA167449	2.86	0.006462
spondin 2, extracellular matrix protein	NM_012445	2.9	0.042509
stanniocalcin 1	NM_003155	2.92	0.080725
endothelial differentiation, sphingolipid G-protein-coupled receptor, 1	NM_001400	2.97	0.00961
T-box 3 (ulnar mammary syndrome)	NM_016569	3	0.00039
phorbol-12-myristate-13-acetate-induced protein 1	NM_021127	3.01	0.03227
Stanniocalcin 1	AW003173	3.08	0.061071
keratin 19	NM_002276	3.11	0.002516
serine (or cysteine) proteinase inhibitor, clade B (ovalbumin), member 7	NM_003784	3.14	0.00069
interleukin 8	NM_000584	3.18	0.101279
interferon-induced protein with tetratricopeptide repeats 3	AI075407	3.18	0.002086
nuclear receptor subfamily 2, group F, member 2	M64497	3.2	0.000033
hypothetical protein FLJ20245	NM_017723	3.22	0.003132
gremlin 1 homolog, cysteine knot superfamily (Xenopus laevis)	NM_013372	3.23	0.008174
Leukocyte-derived arginine aminopeptidase	BE889628	3.29	0.001786
nuclear receptor subfamily 2, group F, member 2	AV703465	3.34	0.001321
CARD only protein	NM_052889	3.34	0.002268
nidogen (enactin)	BF940043	3.37	0.001398
microphthalmia-associated transcription factor	AL117653	3.37	0.005324
gremlin 1 homolog, cysteine knot superfamily (Xenopus laevis)	AF154054	3.38	0.002796

gb:AV646597 /DB_XREF=gi:9867611 /DB_XREF=AV646597 /CLONE=GLCAPB04 /FEA=EST /CNT=57 /TID=Hs.51615.0 /TIER=Stack /STK=34 /UG=Hs.51615 /UG_TITLE=ESTs, Weakly similar to ALU7_HUMAN ALU SUBFAMILY SQ SEQUENCE CONTAMINATION WARNING ENTRY (H.sapiens)	AV646597	3.38	0.0012
5-hydroxytryptamine (serotonin) receptor 2B	NM_000867	3.39	0.033249
nuclear receptor subfamily 2, group F, member 2	AL037401	3.39	0.000131
gb:AV699347 /DB_XREF=gi:10301318 /DB_XREF=AV699347 /CLONE=GKCBPH07 /FEA=EST /CNT=18 /TID=Hs.83623.3 /TIER=Stack /STK=13 /UG=Hs.83623 /LL=9970 /UG_GENE=NR1I3 /UG_TITLE=nuclear receptor subfamily 1, group I, member 3	AV699347	3.5	0.003178
coagulation factor II (thrombin) receptor-like 2	AI378647	3.54	0.003659
RNA binding motif protein 24	AI677701	3.55	0.001578
ST6 (alpha-N-acetyl-neuraminyl-2,3-beta-galactosyl-1,3)-N-acetylgalactosaminide alpha-2,6-sialyltransferase 5 /// ST6 (alpha-N-acetyl-neuraminyl-2,3-beta-galactosyl-1,3)-N-acetylgalactosaminide alpha-2,6-sialyltransferase 5	NM_030965	3.56	0.000061
G protein-coupled receptor 126	AL033377	3.57	0.00181
neurofilament, light polypeptide 68kDa	AL537457	3.58	0.000169
integrin, alpha 6	NM_000210	3.66	0.000904
oxytocin receptor	NM_000916	3.71	0.00264
cathepsin C	NM_001814	3.72	0.00023
neurofilament 3 (150kDa medium)	NM_005382	3.72	0.004978
gb:AA628440 /DB_XREF=gi:2540827 /DB_XREF=af26f02.s1 /CLONE=IMAGE:1032795 /FEA=mRNA /CNT=416 /TID=Hs.83623.2 /TIER=Stack /STK=70 /UG=Hs.83623 /LL=9970 /UG_GENE=NR1I3 /UG_TITLE=nuclear receptor subfamily 1, group I, member 3	AA628440	3.74	0.000585
hypothetical protein FLJ11127	NM_019018	3.9	0.000907
gb:AW263497 /DB_XREF=gi:6640313 /DB_XREF=xn80a06.x1 /CLONE=IMAGE:2700754 /FEA=EST /CNT=6 /TID=Hs.97774.0 /TIER=ConsEnd /STK=3 /UG=Hs.97774 /UG_TITLE=ESTs	AW263497	3.95	0.006195
mannan-binding lectin serine protease 1 (C4/C2 activating component of Ra-reactive factor)	AI274095	3.96	0.000703
homeo box D10	AW299531	3.97	0.004488
response gene to complement 32	NM_014059	4.33	0.003356
forkhead box L2	NM_023067	4.61	0.008719
C-type lectin domain family 2, member B	BC005254	4.62	0.006846
coagulation factor III (thromboplastin, tissue factor)	NM_001993	4.65	0.005223
forkhead box F1	NM_001451	4.65	0.000769
cathepsin C	AI246687	4.93	0
GATA binding protein 2	AL563460	4.98	0.000233
keratin 18	NM_000224	5.02	0.000215

cathepsin C	AI246687	5.1	0.000003
somatostatin receptor 1	R62424	6.12	0.000808
matrix metalloproteinase 1 (interstitial collagenase)	NM_002421	6.7	0.003352
matrix metalloproteinase 3 (stromelysin 1, progelatinase)	NM_002422	6.74	0.006758

Supplementary Table 3. List of genes involved in developmental processes over-expressed in Mb-MSC compares to BM-MSC.

	**Fold increase**
Tbox3	2.97
Gremlin 1	3.3
Homeobox D10	4.13
Nuclear receptor subfamily 2, group F	3.42
Forkhead Box F1	4.3
Agrin	2.14
Oxytocin receptor	3.62
Hes1	2.76

Supplementary Table 4. List of genes involved in adhesion and matrix interaction over-expressed in BM-MSC compare to Mb-MSC

	**Fold increase**
CD24	2.23
Aggrecan	2,27
BMP1	2.1
Cadherin11	2.17
CXCL12	3.11
Connective tissue growth factor	2.3
Integrin alpha11	3.48
Integrin alpha6	
Integrin beta-like 1	3.91
Frizzled	2.03
VCAM1	3.59
Jagged1	2.66

Supplementary Figure 1: Workflow of MSCs isolation

After retrieval of placenta and membrane, tissues were minced into small pieces of less than $1mm^2$ and incubated for 1h in collagenase, hyalluronidase, dispase and DNAse as described in Materials and methods. Mononucleated cells were then isolated using Ficoll gradient. 2 million cells were plated in MSCs media. 1 million of cells were stained for MSCs sorting on SORP FACS Aria 2. Sorted cells were plated on MSCs media. After p=4 all the cells were analyzed for MSCs markers expression and transcriptomic analysis.

Supplementary Figure 2: Comprehensive analysis of MSCs markers on freshly isolated cells from the amniotic membrane.

Cells were stained with mouse anti human CD45-Amcyan, CD34-FITC, CD29-APC-CY7, CD90-AF700, CD73-PE, CD105-AF647.

After FSC-A/SSC-A selection, only $CD45^-$ and $CD34^-$ cells were considered.

We analyzed the expression of the different markers in the freshly isolated cells from the amniotic membrane. We demonstrate that the $CD73^+$ $CD105^+$ population was quite homogenous expressing also CD29 and CD90. The $CD29^-$ $CD90^-$ was also a homogenous population containing mostly $CD73^-$ $CD105^-$ cells. The other cell populations were quite heterogenous in the distribution of the different MSCs markers.

Supplementary Figure 3: Pl/Mb-MSCs constitute a very homogenous population after 4 passages

Exemple of MSCs marker expression of Mb-MSCs. After cell sorting of the different cell populations. We allowed the cells to grow in the MSC media as defined in materials and methods. We then performed after 4 passages flow cytometry analysis of the adherent cells. We can see that there is a quite homogenous population of cells negative for CD45 and CD34 but positive for CD90, CD29, CD73 and CD105. The same results were obtained for all sub-populations sorted previously (data not shown).

Supplementary Figure 4: Immunofluorescence staining of placenta and membrane derived MSCs.

Marker expression of cells derived from placenta and membrane analyzed by FACs were also analyzed by immunofluorescence staining. As in FACs staining cells were positive for CD90, CD29, CD73, CD105, and negative for CD34 and CD45.

Supplementary Table 1: Lists of genes differentially expressed between bone Marrow MSCs and fetal membrane MSCs.

Supplementary Table 2: Lists of genes differentially expressed between bone Marrow MSCs and placenta MSCs.

Supplementary Table 3 . List of genes involved in developmental processes over-expressed in Mb-MSC compares to BM-MSC.

Supplementary Table 4. List of genes involved in adhesion and matrix interaction over-expressed in BM-MSC compare to Mb-MSC.
